# Preparation of Targeted Delivery Materials and Their Application in Animal Production

**DOI:** 10.3390/ani16182901

**Published:** 2026-09-15

**Authors:** Bingfeng Zheng, Yingcheng Gao, Kaisi Hu, Wei Zhang, Wenjie Zhang, Jian Ma

**Affiliations:** 1 College of Coastal Agricultural Sciences, Guangdong Ocean University, Zhanjiang 524088, China; zhengdanhong@stu.gdou.edu.cn (B.Z.); gaoyingcheng@stu.gdou.edu.cn (Y.G.); huks@stu.gdou.edu.cn (K.H.); 2 Key Laboratory of Livestock and Poultry Healthy Breeding Technology in Northwest China, Xinjiang Agricultural Vocational and Technical University, Changji 831100, China; zhangweigsau@163.com

**Keywords:** targeted delivery, carrier, in vitro simulation, microcapsule, animal production

## Abstract

In livestock production, conventional nutrients and bioactive substances are susceptible to degradation during feed processing, storage, and gastrointestinal transit, resulting in low bioavailability and feed conversion efficiency. Targeted delivery technology, which utilizes carriers such as nanoparticles, microcapsules, and hydrogels, can protect these compounds from premature degradation, regulate their release kinetics, and achieve site-specific accumulation via various administration routes, including oral and injectable delivery. This paper introduces the main types of targeted delivery materials and their preparation methods: ionotropic gelation, emulsion solvent evaporation, and nanoprecipitation for nanoparticle fabrication; spray drying, orifice coagulation bath, ionic crosslinking, complex coacervation, and electrospinning for microcapsule production; and ionic crosslinking, gas shear-assisted ionic crosslinking, oil-in-water emulsion combined with calcium ion crosslinking, and enzymatic crosslinking for gel-based system synthesis. Evaluation methods are also described in detail, covering particle size and polydispersity index analysis, morphological characterization, in vitro simulated digestion, and in vivo fluorescence imaging. This paper reviews the applications of this technology in poultry, pigs, and ruminants, with emphasis on its roles in enhancing intestinal health, regulating immune responses, inhibiting pathogenic bacteria, and improving growth performance and product quality, aiming to provide theoretical references for the rational design and application of targeted delivery systems in precision animal nutrition and sustainable livestock production.

## Simple Summary

Targeted delivery materials are carriers, such as nanoparticles, microcapsules, and hydrogels, that protect nutrients and bioactive compounds from degradation during feed processing and digestion, enabling them to be released at specific sites within the animal’s gastrointestinal tract. These materials can be used to improve feed utilization, intestinal health, immune function, growth performance, and product quality in poultry, pigs, and ruminants. This review provides animal nutritionists, feed manufacturers, livestock producers, and veterinary researchers with an additional toolkit for developing more precise and efficient nutritional strategies. However, targeted delivery materials are not a standalone solution, and their effectiveness depends on carrier design, animal physiology, and production goals. Some of the discussed preparation and evaluation techniques are already applied in specialty feed additive production and hold promise for broader integration into precision livestock farming systems.

## 1. Introduction

In modern livestock production, the objectives of nutritional regulation have gradually shifted from satisfying the basal requirements of animals to providing precise, highly efficient, and functional nutrient supply. However, nutrients and bioactive compounds, such as amino acids and fatty acids, are susceptible to degradation during the feed processing, storage, and complex environment of gastrointestinal digestion, which reduces their utilization efficiency. Likewise, proteins in feed can undergo oxidation or Maillard reaction, which further decreases their digestibility and bioavailability [1]. In addition, for ruminants, ruminal microorganisms are primarily involved in the breakdown of proteins, amino acids, polyphenols, and other substances. Because of the rumen’s fermentative activity, a large proportion of unabsorbed nutrients are extensively degraded in advance, preventing active compounds from reaching the intended post-ruminal site [2,3]. Colitis is not restricted to humans, and it is also highly prevalent in production animals such as pigs, cattle, and poultry [4,5,6]. As the livestock sector moves towards reducing antibiotic use, colon-targeted drug delivery has emerged as a key focus in veterinary pharmaceutical development [7]. In the treatment of colitis, the pathological site is located in the hindgut. Conventional therapeutic approaches lack targeting specificity for the inflamed area and are often accompanied by side effects such as hepatotoxicity and infection, thus failing to fundamentally prevent disease recurrence at the source [8,9]. In this context, targeted delivery technology offers a novel solution to improve the stability and effective utilization of nutrients. Originally developed in the pharmaceutical and biotechnology fields, targeted drug delivery systems (TDDS) are designed to release bioactive compounds precisely at the target site in a controlled manner. Compared with conventional delivery methods, TDDS exhibits notable advantages, including high delivery efficiency, prolonged action, reduced effective dosages, and cost savings [10].

With the advancement of TDDS technology in the medical field, the livestock industry has also begun to gradually adopt targeted delivery technologies to improve feed utilization [11]. In production practice, in order to increase the proportion of polyunsaturated fatty acids and reduce the content of saturated fatty acids in livestock products, it is necessary to overcome the extensive ruminal biohydrogenation of polyunsaturated fatty acids in the rumen [12,13]. Besharati et al. [14] reported that dietary supplementation with 7% chitosan-encapsulated flaxseed oil can effectively reduce the extent of biohydrogenation of unsaturated fatty acids in the rumen of dairy cows. After 24 h of in vitro incubation, this treatment increased the proportion of unsaturated fatty acids from 15.08% to 37.88%, while decreasing saturated fatty acids from 84.16% to 61.05% as compared with the control group, thereby improving the milk fatty acid profile. This finding indicates that targeted delivery technology can effectively regulate ruminal fatty acid hydrogenation, which provides a feasible nutritional strategy for improving the fatty acid profile of ruminant products (e.g., milk and meat) through nutritional regulation. But chitosan may exert inhibitory effects on rumen microbial activity or fibre degradation, and its long-term safety and impacts on productive performance require further investigation [15,16]. Targeted delivery technology can effectively modulate ruminal metabolic processes and provides a novel feasible strategy for the protection of rumen-bypass nutrients. The direct translation of targeted delivery technologies from the medical field to animal production still faces considerable challenges.

Feed additives are typically incorporated into the diet at relatively low inclusion levels and administered repeatedly over extended periods. Unlike conventional pharmaceutical agents that are given as discrete therapeutic doses, these additives must also withstand high-temperature pelleting, extrusion, and long-term storage, resulting in sustained dietary exposure rather than acute dosing events. These characteristics, together with marked interspecies differences in gastrointestinal pH, enzyme profiles, and transit time, further preclude the direct cross-species application of existing delivery systems [17,18,19]. In the field of animal nutrition, targeted delivery technology is not merely the encapsulation of target substances; rather, it is a comprehensive strategy that integrates material structure, physiological characteristics of the digestive tract, and nutritional requirements of animals. Through three key steps—protection, transport, and site-specific release—this approach enables nutrients to exert their optimal effects within the animal body [20]. Although some studies have made progress in the preparation and application of targeted materials, there remain relatively few narrative reviews dedicated to the development of specialized carrier materials, the establishment of in vitro and in vivo evaluation systems, and long-term safety assessments. It should be noted that the present review is a narrative review rather than a systematic one, and aims to provide a broad, structured, and critical overview of the current application status of targeted delivery materials in animal production. This paper summarizes the types, preparation techniques, and evaluation methods of targeted delivery materials, and reviews the current application status of these materials in the field of animal nutrition, with the aim of providing a reference for the application of targeted delivery technology in areas such as precision nutrition, green feed additives, and healthy breeding. Importantly, the term ‘targeting’ as used in this review encompasses a broad spectrum of mechanisms. While some systems are designed with active recognition ligands, many carriers—especially nanoparticles—function primarily through passive protection, prolonged gastrointestinal retention, or responsiveness to local pH or enzymatic conditions, without involving specific molecular recognition. Therefore, the delivery performance of each system should be interpreted according to the actual design and mechanism of each system, rather than assuming active targeting by default.

Beyond the material categories systematically covered in this review, a range of alternative delivery platforms, such as liposomes, nanoemulsions, cyclodextrin inclusion complexes, solid lipid nanoparticles (SLNs), nanostructured lipid carriers (NLCs), and conventional rumen-protective coating technologies, have also shown potential for improving nutrient stability and bioavailability [21,22,23]. Lipid-based delivery systems can enhance the protection and utilization efficiency of hydrophobic bioactive compounds, while cyclodextrin complexes improve the solubility and stability of poorly soluble compounds [24,25,26]. In ruminants, lipid- and polymer-based coating technologies have been applied to reduce ruminal nutrient degradation and improve intestinal availability [27,28]. However, these platforms are not systematically discussed in the present review because their application in commercial livestock production remains limited by challenges related to production costs, large-scale manufacturing, stability during feed processing, and insufficient in vivo validation under practical feeding conditions [24,29].

## 2. Methods

As this is a narrative review, the literature search was designed to be comprehensive rather than systematic, aiming to capture a broad spectrum of available evidence on targeted delivery systems, carriers, in vitro simulation, microcapsules, and their applications in animal production. Relevant literature was retrieved from the following electronic databases: Web of Science, ScienceDirect, PubMed, Scopus, and the Google Scholar search engine. The search was restricted to peer-reviewed journals and conference proceedings, and the document types were limited to original research articles, reviews, and conference papers. The language was restricted to English, and the search timeframe was focused primarily on publications between 2016 and 2026 to ensure the inclusion of recent advances.

To enhance transparency and reproducibility, a core Boolean search string was applied across all databases: (targeted delivery OR carrier OR microcapsule OR encapsulation) AND (in vitro simulation OR digestion OR release) AND (animal production OR livestock OR poultry OR ruminant). This core string was subsequently adapted for each specific database to accommodate its unique syntax and search engine functionalities. For example, adjustments included the use of appropriate truncation symbols (e.g., “*” for Web of Science and Scopus, or “$” for PubMed), field tags (e.g., [TIAB] for PubMed, or “Article Title” for Scopus), and proximity operators (e.g., “NEAR” or “ADJn”) to optimize the sensitivity and specificity of the retrieval process. When necessary, this primary search was supplemented with additional keyword combinations, including but not limited to colon-targeted, pH-responsive, controlled release, gastrointestinal tract, and feed additive, to capture relevant literature that might not have been identified by the core string alone.

The initial search across all databases yielded 376 records after the removal of duplicates. These records were first screened by title and abstract against predefined inclusion criteria, which required relevance to targeted delivery in the context of animal nutrition or production, the use of in vitro models, and the provision of original experimental or mechanistic insights. Following this initial screening, 182 records were considered potentially relevant and were advanced to full-text assessment. After a thorough full-text evaluation, 141 of these 182 records were ultimately retained for final inclusion in this review.

## 3. Types and Preparation Methods of Targeted Delivery Materials

### 3.1. Nanoparticle

To ensure that nutrients reach the designated sites along the digestive tract or specific tissues and organs efficiently and stably, and achieve precise release, the key lies in the design of delivery carriers. These carriers are generally required to possess good biocompatibility, low toxicity, biodegradability, controlled release characteristics, and high targeting efficiency [30,31,32]. Nanoparticles are particles with sizes ranging from 1 to 100 nm, characterized by a large specific surface area and strong permeability, and are widely used to encapsulate nutrients and feed additives, as well as in feed processing [33,34,35]. Importantly, the use of nanoparticles as delivery carriers should not be equated with active targeting. Depending on their physicochemical properties and structural design, nanoparticles may primarily function by protecting encapsulated compounds from degradation, delaying their release, increasing residence time or mucosal contact, or responding to specific gastrointestinal environmental stimuli such as changes in pH, enzyme activity, or microbial conditions [36,37,38]. These mechanisms represent passive protection, passive localization, and controlled or stimuli-responsive release rather than active targeting per se. In contrast, active targeting requires the deliberate incorporation of targeting ligands or recognition elements onto the nanoparticle surface to facilitate specific interactions with receptors or other molecular targets at the intended site or on target cells [39,40]. Therefore, in the context of animal nutrition, nanoparticle-based delivery should be considered a broad carrier strategy. Its functions encompass passive protection mechanisms—including site-dependent localization, environmentally triggered release, rumen protection, and controlled release—as well as, in selected systems, active targeting mediated by ligands or receptors. These mechanisms may operate simultaneously within a single delivery system, but they should be distinguished according to their underlying mechanisms rather than collectively described as “targeting”. Although nanoparticles are conventionally defined as particulate materials with at least one dimension below 100 nm, it should be recognized that the literature frequently applies the term ‘nanoparticles’ to systems with mean diameters exceeding this threshold [41,42]. The precise boundary between nano- and microparticles therefore remains operationally ambiguous and subject to ongoing debate. Toragall et al. [43] prepared lutein nanoparticles using the ionotropic gelation method. Positively charged chitosan was dissolved in acetic acid, and lutein was dissolved in oleic acid, followed by the addition of polysorbate with thorough mixing. Subsequently, a negatively charged sodium alginate aqueous solution was added to the mixture, and the resulting system was subjected to ice-bath sonication and high-speed homogenisation to form encapsulated nanoparticles. This encapsulation strategy is of considerable significance in animal nutrition, as it can effectively improve the aqueous dispersibility, physicochemical stability, and gastrointestinal tolerance of lutein, thereby enhancing its oral bioavailability and facilitating targeted delivery to intestinal absorption sites [44]. This method avoids the use of organic solvents and high temperatures, thus preserving the structural integrity and bioactivity of the bioactive compound to the greatest extent possible, offering a practical solution to overcome the inherent limitations of free lutein, such as poor solubility and susceptibility to degradation during feed processing and digestion [45].

**Figure 1:**
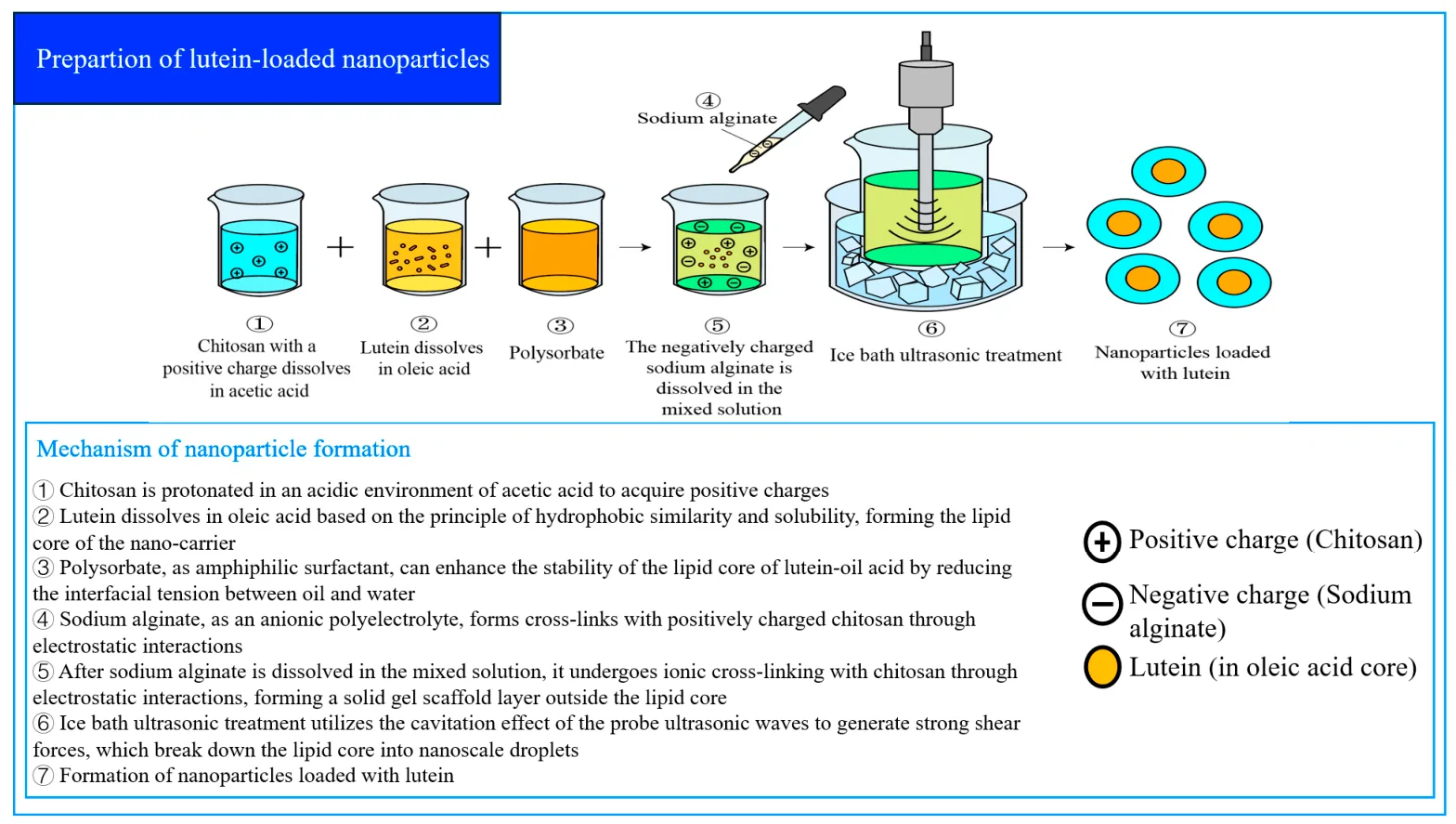
Preparation process of lutein-loaded nanoparticles [43].

Although the ionotropic gelation method is applicable for crosslinking encapsulation of cationic polymers (e.g., chitosan) and anionic polysaccharides (e.g., sodium alginate), it is difficult to achieve high encapsulation efficiency for short-chain fatty acids (SCFA) that are highly water-soluble or weakly acidic. To address this, Carvalho et al. [46] prepared nanoparticles loaded with butyrate and propionate using the emulsion solvent evaporation method. After preparing the aqueous and oil phases, the oil phase was added dropwise to the aqueous phase under ice-bath conditions, and the mixture was treated with an ultrasonic homogeniser to form an emulsion. The emulsion was then subjected to magnetic stirring to evaporate the organic solvent, yielding the nanoparticles. The emulsion solvent evaporation method can effectively encapsulate SCFA; however, the resulting nanoparticles are typically present as colloidal suspensions, which are prone to sedimentation upon prolonged storage and exhibit poor stability during animal feed processing. Additionally, issues related to solvent residues and the feasibility of this process for industrial production need to be considered. Therefore, further encapsulation of these nanoparticles into micron-scale carriers is required to improve their stability and facilitate industrial application. Spindler et al. [47] employed a spray-drying method to assist in the preparation of poly(lactic-co-glycolic acid) (PLGA) nanoparticles. The pre-prepared nanoparticles were mixed with a chitosan solution at a certain mass ratio, and the nanoparticles were successfully embedded into the chitosan microsphere matrix using a spray dryer. This technique offers high processing efficiency and is easily scalable, and the micron-scale matrix significantly improves particle stability during storage and feed processing. It should be noted that the high-temperature airflow may inactivate thermosensitive substances; therefore, the thermal stability of the targeted bioactive compounds should be carefully evaluated prior to industrial application, and the uniformity of nanoparticle distribution within the microspheres is difficult to precisely control. In addition to the aforementioned methods, nanoprecipitation is another approach for the preparation of nanoparticles. In this method, an organic phase containing hydrophobic active substances is rapidly injected into an aqueous phase, where solvent diffusion induces a supersaturation state, leading to the spontaneous formation of nanoparticles [48]. This technique is extremely simple to operate; however, it is primarily suitable for encapsulating hydrophobic substances and suffers from the issue of residual organic solvents [49]. Ionotropic gelation is the most commonly employed method for preparing nanoparticles used to encapsulate active substances. Figure 1 illustrates the complete process for preparing lutein-loaded nanoparticles via ionotropic gelation. In summary, nanoparticles prepared by various methods can improve their stability in the complex digestive tract, achieve efficient encapsulation of nutrients and bioactive factors, and enhance nutrient utilization, thereby laying a foundation for the application of nanoparticles in animal production.

Different nanoparticle preparation methods exhibit notable differences in encapsulation efficiency, applicability to active substances, process complexity, and scalability for industrial application. Ionotropic gelation, which typically forms nanostructures through electrostatic interactions between oppositely charged polymers, offers advantages such as mild preparation conditions, simple operation, and good biocompatibility, making it suitable for encapsulating polysaccharide- and protein-based materials [50]. This method is highly dependent on the charge properties of polymers and the solubility of the core material, resulting in limited encapsulation efficiency for water-soluble small-molecule active substances. The resulting nano-dispersions may suffer from insufficient stability during long-term storage [35]. Emulsion solvent evaporation can form nanoparticles at the oil–water interface, making it suitable for encapsulating hydrophobic or weakly polar active substances, and enables control over particle size and drug loading by adjusting emulsification conditions [46]. This method involves the use of organic solvents, which adds safety control requirements and cost burdens during production; continuous production and solvent recovery remain significant constraints for large-scale application. Spray drying benefits from well-established industrial equipment infrastructure and can convert nano-dispersions into powder forms that are easy to store and transport, facilitating their integration with feed processing systems [51]. However, high temperatures and thermal stress may affect the stability of heat-sensitive active substances, thus typically necessitating the addition of protective agents or optimization of drying conditions [52]. In animal nutrition applications, the selection of nanoparticle preparation methods should not be based solely on encapsulation efficiency, but also require comprehensive consideration of the properties of the active substances, target release sites, production costs, storage stability, and feed processing compatibility [53]. Hydrophobic compounds favour emulsion-based techniques, whereas hydrophilic ones are better suited to ionotropic gelation. Rumen protection and colon targeting demand distinct carrier responsiveness. Scalability and resistance to pelleting stresses are critical for industrial translation. Thus, method selection demands a balanced, multi-factor evaluation.

Despite considerable advances in targeted delivery materials, successful encapsulation does not always guarantee improved biological performance. Several studies have reported limitations associated with carrier instability, insufficient drug release, or unexpected biological responses. For example, nanoparticle-based delivery systems may suffer from aggregation, premature cargo leakage, or loss of biological activity during storage and gastrointestinal transit. In lipid nanoparticle systems, chemical interactions between carrier components and encapsulated molecules have been shown to generate degradation products that compromise cargo activity, highlighting the importance of evaluating long-term formulation stability rather than only initial encapsulation efficiency [54]. It should also be noted that excessive reliance on carrier materials may introduce additional biological burdens, as non-degradable or slowly degradable materials can accumulate in tissues and potentially induce inflammatory responses or toxicity [55]. The design of targeted delivery systems for animal production should consider not only encapsulation efficiency and controlled release characteristics but also degradation behaviour, biological compatibility, and long-term safety. Taken together, the various nanoparticle preparation techniques currently available each have their own scope of application and limitations, and a universal approach that accommodates all application scenarios remains lacking. In practice, the selection of an appropriate method should be based on the physicochemical properties of the active compound, the targeted delivery objectives, and the requirements of feed processing technology. Meanwhile, enabling nanoparticles to efficiently cross the gastric acid and mucus barriers and subsequently be absorbed by intestinal epithelial cells remains a core challenge in this field. The preparation methods of nanoparticles, the encapsulated substances, and the comparison of their advantages and disadvantages are shown in Table 1.

### 3.2. Microcapsule

Microcapsules consist of a dense shell and a core, whose physical state is not necessarily liquid but may also be semi-solid or solid, depending on the core material and preparation method. The physical barrier provided by the shell membrane effectively protects the internal active substances, while the liquid core enables on-demand release and efficient loading [56]. Microcapsules typically range in size from 1 to 100 µm. Their targeted delivery mechanism relies on the responsiveness of the shell material, which enables the controlled release of encapsulated active ingredients at designated sites [57,58]. The most common approach is environmentally responsive targeting, which exploits the specific pH conditions of the gastrointestinal tract to design pH-responsive polymer shells. These shells release the active substances after the microcapsules reach the designated sites, thereby achieving targeted delivery [59]. For example, substantial differences in pH exist among different segments of the gastrointestinal tract. In cattle, reported pH values were approximately 5.4–6.6 in the rumen, 2.3–4.2 in the abomasum, 5.8–6.8 in the small intestine and 5.0–6.8 in the colon, although these values vary with diet and physiological conditions [29]. These distinct pH gradients enable the rational design of carriers that release their cargo at specific sites according to the local pH.

Various preparation methods are available for microcapsules, including the orifice coagulation bath method, spray drying, ionic crosslinking, and electrospinning techniques. Each method exhibits distinct characteristics in terms of encapsulation efficiency, particle size control, substrate applicability, and industrial feasibility, and the selection should be based on the physicochemical properties of the active substance and the intended application scenario. Zhao et al. [60] prepared microcapsules using the orifice coagulation bath method, selecting sodium alginate and chitosan as composite wall materials and plant essential oils as the core material. After mixing and emulsifying sodium alginate with plant essential oils, polysorbate, and glycerol, the mixture was dropped through a 0.7 mm needle into a coagulation bath containing chitosan and calcium chloride. After magnetic stirring, the microcapsules were obtained through washing, filtration, and drying. The orifice coagulation bath method is simple to operate, but the resulting microcapsules typically have a single-layer wall structure, and probiotics require milder encapsulation conditions during the preparation process. This method is characterized by simple operation, low equipment requirements, and the avoidance of high temperatures and organic solvents, making it suitable for encapsulating volatile or thermosensitive substances such as plant essential oils, but its limitations include the typically single-layer wall structure of the resulting microcapsules, which affords limited protection for viable microorganisms such as probiotics, and the difficulty in precisely controlling droplet size uniformity, which hampers quality control in large-scale production [61,62,63]. The embedding conditions for probiotics require relatively mild conditions. Ding et al. [64] used an ionic crosslinking method to prepare multilayer calcium alginate composite microcapsules for intestinal targeted delivery of probiotics. Briefly, nanoparticles were first prepared via chemical crosslinking. The probiotic suspension was mixed with the internal aqueous phase containing probiotic protectants, and a water-in-oil-in-water (W/O/W) double emulsion was formed by homogenization. The probiotic-loaded double emulsion was then mixed with sodium alginate solution and the antacid calcium carbonate, and the mixture was extruded through a syringe into calcium chloride solution to form calcium alginate hydrogel microspheres, ultimately yielding microcapsules with a multilayer embedded structure. The chemical crosslinking method imposes high requirements on shear force and ionic strength for the encapsulated materials and is not suitable for easily oxidized lipid-based active substances, and involves cumbersome procedures that render large scale production challenging [65]. Additionally, the oil phase and surfactants employed in the W/O/W double emulsion system may leave undesirable residues in the final microcapsule products. Such residual components not only raise biosafety concerns but also necessitate extensive washing and purification steps, further increasing process complexity and overall production costs, and thereby posing additional barriers to industrial-scale manufacturing [66,67].

In contrast, the complex coacervation method can utilize oppositely charged polymers to spontaneously form coacervate layers coating the encapsulants through electrostatic interactions under aqueous conditions at room temperature. Chen et al. [68] prepared walnut oil microcapsules using the complex coacervation method. Sodium alginate solution was thoroughly mixed with walnut oil, followed by the addition of chitosan solution. The pH was adjusted to trigger electrostatic interactions between positively charged chitosan and negatively charged sodium alginate, forming a complex coacervate layer on the surface of the oil droplets. Subsequently, calcium chloride solution was added for curing, and the mixture was allowed to stand in an ice bath, ultimately yielding microcapsule powder via freeze-drying. This method operates under mild conditions without organic solvents or high temperature treatment, effectively protecting unsaturated fatty acids from oxidation [69]. Although the above study focused on a food application, this encapsulation strategy holds considerable promise for animal nutrition, particularly for the protection of polyunsaturated fatty acids (PUFAs) in feed additives against oxidative deterioration during storage and feed processing, thereby improving the nutritional quality of animal products (e.g., meat and milk). Its drawbacks include the susceptibility of the coacervation process to multiple factors such as pH, ionic strength, and polymer concentration, resulting in stringent operational conditions and poor reproducibility. Different microcapsule preparation methods each have their own advantages and disadvantages, and are suitable for encapsulation requirements of various types of active substances, thereby offering additional optional strategies for the targeted delivery of nutrients.

In addition to the methods described above, electrospinning technology provides a novel approach for microcapsule preparation. This technique uses a high-voltage electric field to jet a polymer solution containing core and wall materials, overcoming the surface tension of the solution under the action of electric field force to form nanofibrous or microfibrous structures or microcapsules [70]. Compared with conventional methods, electrospinning enables one-step fabrication of fibrous microcapsules with a high specific surface area, and allows flexible control of carrier size and morphology by adjusting parameters such as voltage, flow rate, and collection distance. However, this technique is currently limited mainly to water-soluble polymers (e.g., polyvinyl alcohol, chitosan) as wall materials, with restricted applicability to hydrophobic wall materials, and issues of solvent residue and scale-up production remain to be resolved. Chang et al. [71] utilized electrospinning to prepare nanofibrous microcapsules encapsulating lactic acid bacteria. In an in vitro simulated rumen–abomasum–intestinal environment, the microcapsules maintained structural integrity in rumen fluid, while the release of lactic acid bacteria in the intestinal phase was increased by threefold, demonstrating that electrospun microcapsules can successfully protect lactic acid bacteria through the rumen and achieve targeted release in the intestine. This finding highlights the potential of electrospinning in the application of gastrointestinal delivery.

In summary, microcapsule preparation methods exhibit notable differences in encapsulation mechanisms, structural integrity, particle size control capability, applicability to core materials, and potential for large-scale application. The orifice coagulation bath method is simple to operate and requires low equipment investment, forming gel microcapsules by dropping polymer solutions into a coagulation bath, making it suitable for natural polysaccharide wall materials such as alginate. However, the traditional dropping process typically produces single-layer structures, and particle size uniformity is easily influenced by nozzle diameter, dropping speed, and gelling conditions, while precise control over core material release behavior remains relatively limited [72]. Ionic crosslinking can form stable networks through interactions between polyelectrolytes and multivalent ions, and can be combined with layer-by-layer assembly strategies to construct multi-layered protective structures, thus offering advantages in protecting environmentally sensitive core materials such as probiotics [73]. This method is sensitive to ionic strength, polymer properties, and processing conditions, and the construction of multi-layered structures increases preparation complexity and production cycle time; for hydrophobic or easily oxidizable lipid-based substances, it is typically necessary to combine emulsification techniques or composite wall material systems to further improve encapsulation efficiency [74]. Complex coacervation utilizes electrostatic interactions between oppositely charged polymers to form a coacervate phase, offering the advantages of mild conditions and no requirement for high-temperature treatment or organic solvents, making it suitable for encapsulating heat- and oxygen-sensitive active substances such as vegetable oils and essential oils [75]. This process is highly dependent on factors such as pH, ionic strength, and polymer concentration, with a narrow processing window, and maintaining consistent product properties during process scale-up remains to be further explored. Overall, the orifice coagulation bath method, owing to its simple process and low equipment requirements, shows potential for application in the development of low-cost functional feed additives. Ionic crosslinking is suitable for delivery systems requiring high protection of functional ingredients but requires further optimization of production efficiency. Complex coacervation offers advantages in protecting lipid-soluble active substances, but its industrial application still needs to address process control issues [17,76,77]. In the future, the selection of microcapsule preparation strategies should comprehensively consider core material properties, target release sites, production costs, storage stability, and feed processing compatibility.

The aforementioned microcapsule preparation methods each have their own advantages and disadvantages in terms of applicable core materials, protective efficacy, process complexity, and scalability potential. The orifice coagulation bath method is simple and rapid, but offers limited protection for the encapsulated cargo [78]; ionic crosslinking provides strong protection but involves laborious procedures [50]; complex coacervation operates under mild conditions but is sensitive to environmental factors [79]; and electrospinning technology allows flexible modulation of carrier morphology, yet large-scale production remains immature [80]. In practical research, the choice between protection efficiency and process feasibility depends primarily on core material properties—namely, thermosensitivity, oxidative susceptibility, viability requirements of live microorganisms, and the intended release site. The preparation method of microcapsules, the encapsulated substances, as well as the comparisons of their advantages and disadvantages are presented in Table 2.

### 3.3. Gel

A gel is a gelatinous and elastic material formed by a three-dimensional crosslinked polymer network swollen in a solvent. The stiffness of the gel network can range from soft to rigid, depending primarily on the physical and chemical forces between polymer chains within the matrix, such as π–π stacking, hydrogen bonding, metal coordination, hydrophobic interactions, and van der Waals forces. These interactions endow gels with reversible and stimuli-responsive properties [81,82,83]. The size of nanogels typically ranges from 20 to 200 nm [84]. Nanogels can function as targeted delivery carriers, delivering active substances to designated sites and achieving controlled release. Various preparation methods are available, each with distinct characteristics in terms of particle size control, gelation mechanisms, and substrate applicability. Ionic gelation is one of the most widely adopted approaches for nanogel preparation, based on the electrostatic interaction between oppositely charged polymers to spontaneously form polyelectrolyte complexes under mild conditions. Luo et al. [85] prepared composite nanogels using the ionotropic gelation method. Chitosan was dissolved in pure water, and a NaOH solution containing enrofloxacin was added to form the inner core. Meanwhile, sodium alginate solution and CaCl_2_ solution were mixed to form the shell gel. The nanoparticles were then introduced into the sodium alginate-calcium ion system, and composite nanogels with a core–shell structure were formed through electrostatic interactions. This method is simple to operate, obviates the need for organic solvents or high temperatures, and is suitable for encapsulating water-soluble substances and proteins [86]. But the reaction typically proceeds through rapid solution mixing, resulting in limited controllability over gel particle size and structure, and making it challenging to obtain nanogels with good monodispersity [87,88].

Given that enrofloxacin is a veterinary antibiotic, its residual presence in edible tissues and the associated withdrawal period must be carefully considered when applying this nanogel formulation in food-producing animals [89]. The altered pharmacokinetic and tissue distribution profiles resulting from nano-encapsulation may affect the elimination kinetics of the drug, thereby influencing the required withdrawal time before slaughter or milk collection [90,91]; therefore, comprehensive residue depletion studies are essential to establish a safe withdrawal period for the nano-formulated product. To achieve more finer control over gel size, Wang et al. [92] employed a gas shear-assisted ionic crosslinking method to prepare fullerene phenol-loaded gel microspheres. Briefly, fullerene phenol nanoparticles were first dispersed in an aqueous pectin solution, and the mixture was then extruded through a coaxial needle, where the shear force generated by injected N_2_ fragmented the stream into uniform droplets. After collection, the droplets were added to a solution containing zinc acetate and chitosan. Pectin and Zn^2+^ formed gel microspheres through ionic crosslinking, and chitosan was subsequently grafted onto the surface of the microspheres, yielding gel microspheres with colon-targeting functionality. The shear-assisted ionic crosslinking method requires the use of a coaxial needle and N_2_, which imposes relatively high equipment demands. This approach achieves droplet homogenization through air-shear, yielding a narrow particle size distribution and good reproducibility, and can be performed at ambient temperature. However, it necessitates relatively sophisticated equipment such as coaxial needles and airflow control systems, leading to higher operational thresholds and costs. Consequently, although promising for laboratory-scale studies, this technique may not be practically viable for large-scale industrial production.

To streamline the preparation process, calcium ion crosslinking can serve as an alternative for gel preparation. Yuan et al. [93] prepared low-methoxyl pectin microgels using a water-in-oil emulsion combined with calcium ion crosslinking. An aqueous phase consisting of low-methoxyl pectin solution and milk protein nanotubes was mixed with an oil phase containing a non-ionic surfactant to form a water-in-oil emulsion through emulsification. CaCl_2_ solution was then introduced to induce crosslinking, followed by vacuum filtration and washing with ethanol to remove the oil phase, yielding the microgels. This method uses emulsion droplets as micro-reactors, allowing gelation to proceed at room temperature with simple equipment, and is suitable for encapsulating bioactive substances that are sensitive to chemical reagents. The procedure involves multiple steps, and residual oil and surfactants may compromise the biosafety of the final product. Enzymatic crosslinking provides a green and efficient alternative for gel preparation. This method employs transglutaminase to catalyze crosslinking reactions between glutamine and lysine residues in protein molecules, forming a stable three-dimensional network under mild conditions [94]. Taking the gelatin–PVA composite system as an illustration, Corona-Escalera et al. [95] used microbial transglutaminase (mTGase) to crosslink gelatin with polyvinyl alcohol (PVA), producing a composite hydrogel loaded with probiotics. The results demonstrated that the hydrogel achieved a probiotic encapsulation efficiency exceeding 90% and a freeze-drying survival rate of 91%. In simulated gastric fluid, the hydrogel maintained structural integrity with probiotic survival above 94%, while in intestinal fluid the hydrogel completely disintegrated to release probiotics, and the material displayed no cytotoxicity toward HT29 intestinal epithelial cells. This method operates under mild conditions without organic solvents or high temperatures, is biocompatible with bioactive substances, and offers high encapsulation efficiency and outstanding protective effects. However, the procedure is relatively elaborate, involving multiple steps including enzyme solution preparation, crosslinking reaction, enzyme inactivation, and freeze-drying, and the use of enzymes escalates costs, leaving large-scale production still to be further explored.

A comprehensive comparison of the aforementioned gel preparation methods reveals substantial differences among the various techniques in terms of gelation mechanisms, size controllability, process complexity, scalability, and applicable scenarios. Ionotropic gelation typically relies on ionic interactions between multivalent ions and charged polymer chains to form three-dimensional network structures [33]. This method offers advantages such as simple operation, mild conditions, and no requirement for organic solvents or high-temperature treatment, making it one of the most widely applied approaches for polysaccharide-based gel preparation [96]. Conventional ionotropic gelation processes exhibit relatively rapid gelation kinetics, which complicates precise control over network structure and particle size uniformity; At the same time, water-soluble active substances may suffer from diffusion loss during gel formation, thereby diminishing encapsulation efficiency [97]. Gas shear-assisted ionic crosslinking introduces gas shear forces to the conventional ionotropic gelation process, improving particle size uniformity by adjusting airflow velocity and droplet formation dynamics, making it suitable for delivery systems with stringent size control requirements [98]. Nevertheless, this method necessitates additional gas control devices and specialized jetting structures, entailing higher equipment demands, and shear parameters still require optimization for different material systems. The water-in-oil emulsion combined with calcium ion crosslinking method involves dispersing followed by induction of an aqueous phase containing polymers and crosslinking agents into an oil phase to form an emulsion, followed by induction of gel network formation; particle size can be controlled by adjusting emulsion composition and emulsification conditions, and this approach holds promise for continuous preparation [93]. But this method involves an oil phase and emulsifier system, requiring subsequent washing and purification steps, which increases process complexity and may affect product safety evaluation. From the perspective of scalable application, ionotropic gelation benefits from mature technology and simple equipment, although particle size consistency in large-scale production still requires further optimization; the gas shear-assisted method offers good size control capability and is suitable for pilot-scale preparation; the emulsion crosslinking method possesses potential for continuous production, although issues related to emulsifier removal and process control remain unresolved. From an economic standpoint, ionotropic gelation generally entails lower equipment investment and production costs, whereas the gas shear-assisted and emulsion crosslinking methods entail relatively higher overall production costs due to additional equipment or post-treatment steps. Therefore, in the field of animal nutrition, the selection of gel preparation methods should comprehensively consider the properties of the active substances, target release sites, size requirements, production costs, and scalability needs. The preparation method of the gels, the encapsulated substances, as well as the comparisons of their advantages and disadvantages are presented in Table 3.

Although the preparation methods described above have demonstrated promising encapsulation efficiency and controlled-release characteristics under laboratory conditions, their translation into commercial feed production remains challenging. During feed manufacturing, delivery systems are subjected to multiple stresses, including thermal treatment, mechanical shear, moisture fluctuations, and prolonged storage. Processes such as pelleting and extrusion may disrupt carrier structures, compromise encapsulation efficiency, or accelerate the degradation of sensitive bioactive compounds. Previous studies have emphasized that the stability of encapsulated probiotics and bioactive compounds during processing and storage is highly dependent on the encapsulation materials, preparation techniques, and processing conditions [99]. Despite these advances, many targeted delivery systems developed at laboratory scale have not yet been systematically evaluated under realistic feed-processing conditions. Their stability during pelleting, extrusion, mixing, transportation, and long-term storage remains insufficiently investigated. Furthermore, large-scale manufacturing feasibility, production cost, compatibility with existing feed-processing facilities, and regulatory requirements represent important factors constraining commercial application. Therefore, future research should focus not only on improving gastrointestinal targeting efficiency but also on developing robust delivery systems capable of maintaining functional stability throughout the entire feed production and storage chain.

In the transition from laboratory-scale preparation to industrial production, comprehensive consideration must still be given to production costs, process continuity, and scale-up capacity [100]. Among the current preparation technologies, spray drying is regarded as possessing high industrial application potential due to its continuous operation, high throughput, and favorable compatibility with existing food and feed processing streams [101,102]. In contrast, although ionotropic gelation and orifice coagulation bath methods offer mild preparation conditions and good biocompatibility, making them suitable for encapsulating polysaccharide-based materials and heat-sensitive active substances, their relatively low production efficiency and difficulties in controlling particle uniformity limit their further scale-up applications [103]. Furthermore, some advanced preparation strategies, such as gas shear-assisted ionic crosslinking and water-in-oil-in-water (W/O/W) double emulsion methods, can achieve precise size control and construction of complex structures; but their demanding equipment requirements and high process complexity may lead to substantially increased production costs [65]. Therefore, future research should further optimize continuous manufacturing processes and develop novel large-scale production strategies that balance delivery performance with cost-effectiveness.

**Figure 2:**
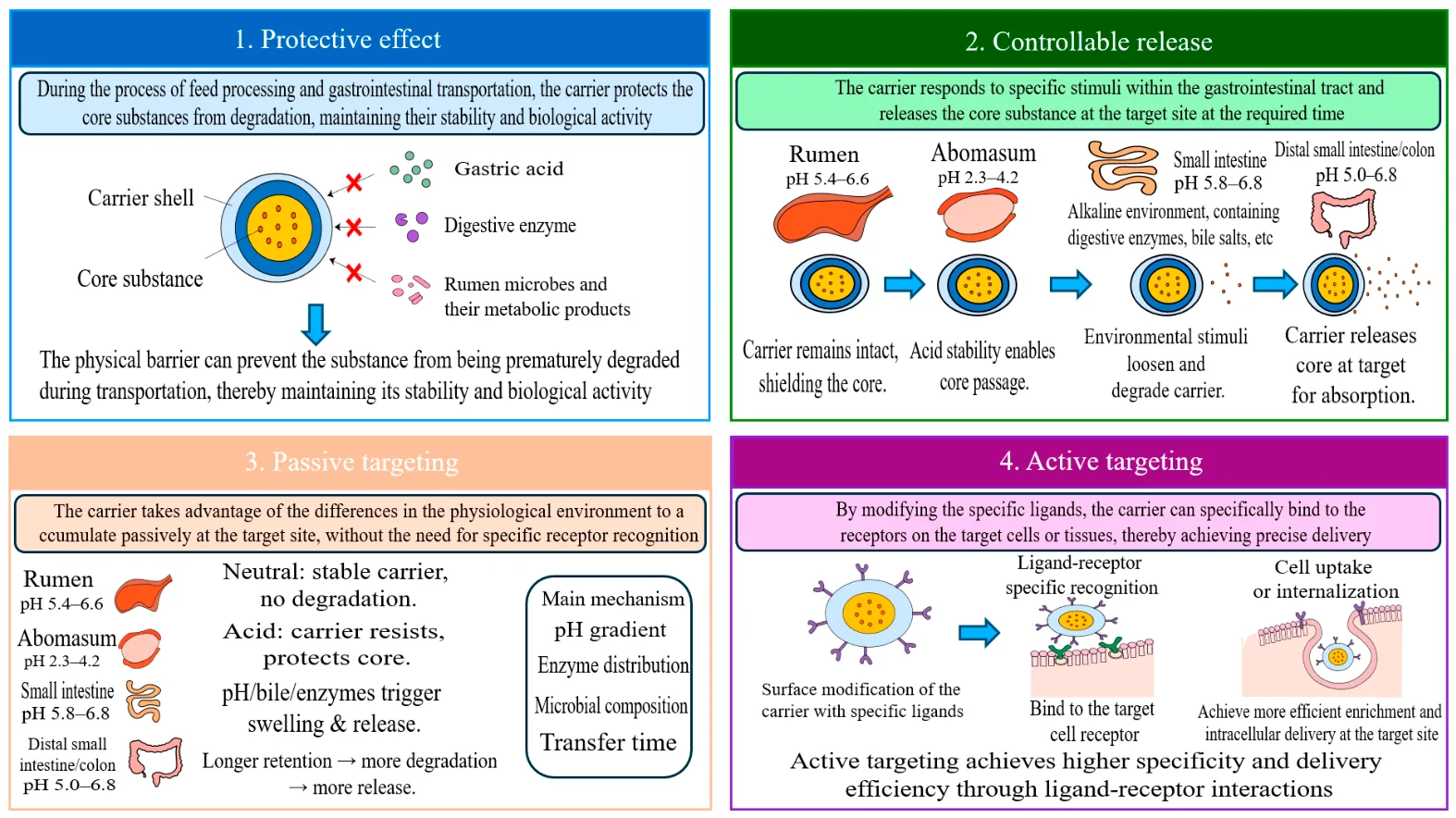
Schematic illustration of the key mechanisms of targeted delivery carriers in the gastrointestinal tract: protective effect, controllable release, passive targeting, and active targeting.

In summary, for nanoparticles, the ionotropic gelation method is suitable for chitosan/sodium alginate crosslinking encapsulation, the emulsion solvent evaporation method is appropriate for encapsulating short-chain fatty acids, and spray drying can enhance the stability of nanoparticles. In addition, the nanoprecipitation method utilizes solvent displacement co-precipitation to form nanoparticles loaded with hydrophobic substances. For microcapsules, the orifice coagulation bath method is simple to operate, the ionic crosslinking method can construct multilayer encapsulation structures, the complex coacervation method is suitable for encapsulating sensitive substances such as vegetable oils, and the extrusion method, with its mild conditions, is appropriate for encapsulating viable microorganisms. As for gels, which are three-dimensional crosslinked networks, the gas shear-assisted ionic crosslinking method enables precise size control, the water-in-oil emulsion combined with calcium ion crosslinking can streamline the preparation process, and the electrospray method, which utilizes a high-voltage electric field and a receiving liquid to form gel microspheres, is suitable for encapsulating bioactive substances that are highly sensitive to chemical reagents. The preparation method of the gel, the encapsulated substances, as well as the comparisons of their advantages and disadvantages are presented in Table 3. The types, structures, materials, and release mechanisms of targeted delivery carriers are compared as shown in Table 4. Schematic diagram of the key mechanisms of gastrointestinal targeted delivery carriers: protective effect, controlled release, passive targeting and active targeting are shown in Figure 2.

## 4. Evaluation Methods for Targeted Delivery Materials

### 4.1. Particle Size, Distribution, and Morphology

In targeted delivery technology, the particle size, size distribution, and morphology of the materials are critically important, as they govern whether the targeted delivery carriers can be successfully transported to the target sites. Particle size influences the ability of carriers to cross biological barriers. The size distribution is evaluated by the polydispersity index (PDI); a lower PDI value indicates a more uniform size distribution and better homogeneity of carrier dimensions, which substantially affects experimental outcomes and reproducibility. Morphology, in turn, affects the flow behaviour of carriers in vivo, their interactions with cells, and the release profile of the encapsulated cargo [104,105,106]. Bao et al. [107] prepared *SNX10*-shRNA plasmid nanoparticles and used a laser particle size analyzer to determine the particle size and size distribution, while scanning electron microscopy was employed to observe nanoparticle morphology. The results revealed that the nanoparticles could precisely target the colon and protect the nucleic acids from gastrointestinal degradation. Overexpression of the *SNX10* gene was found to promote exacerbation of inflammation, whereas administration of the nanoparticles in mice effectively suppressed *SNX10* mRNA expression and reduced the levels of the inflammatory cytokines IL-1β and TNF-α. Although the example originates from the biomedical field rather than direct livestock production, its methodological approach serves as a useful reference for particle size and morphology characterisation in targeted delivery systems. Compared with the nanoscale structure of nanoparticles, the microscale structure of microcapsules is more suitable for encapsulating active substances. Bhagat et al. [108] prepared pH-responsive sodium alginate microcapsules encapsulating six types of nanominerals. Transmission electron microscopy was used to observe the morphology and particle size of the nanominerals, while scanning electron microscopy was employed to examine the surface morphology and structure of the microcapsules. The nanominerals appeared spherical in shape, and the microcapsules exhibited an intact spherical structure, remaining stable in acidic gastric fluid to effectively protect the nanominerals from degradation, while rapidly disintegrating and releasing them in intestinal fluid, thereby achieving targeted delivery of nanominerals to the small intestine. Following oral administration of these microcapsules to rats, the concentrations of mineral elements such as zinc, copper, and manganese in the blood were elevated, and the expression levels of SOD1 and SOD2 enzymes in the liver and kidneys were enhanced. In summary, particle size, distribution, and morphology constitute key physicochemical evaluation metrics for targeted delivery carriers, ensuring stable transport of carriers within the animal body and release at designated targets, and represent the fundamental evaluation indicators for achieving targeted delivery.

### 4.2. In Vitro Simulation

In vitro simulation can replicate the pH conditions and enzymatic activities of the animal digestive tract, thereby enabling assessment of the protective effect of targeted delivery materials on encapsulated compounds and their release efficiency at the target sites, and determining whether they can achieve sustained release and targeted functions. Most targeted delivery materials are administered orally and must traverse the animal digestive tract. The highly acidic environment of the stomach, coupled with the abundant pepsin, can degrade protein- or peptide-based bioactive components and disrupt the structure of the delivery carriers. Therefore, establishing a reliable in vitro simulated gastric fluid evaluation system is of critical importance. Altermann et al. [109] prepared polyhydroxybutyrate nanoparticles modified with the archaeal virus lytic enzyme PeiR. Through the action of PhaC enzyme, PeiR was conjugated onto the nanoparticle surface. PeiR belongs to the C39 peptidase family and is capable of specifically hydrolysing pseudomurein in the cell walls of methanogenic archaea, thereby lysing methanogenic cells predominantly of the genus *Methanobrevibacter*. After incubating the nanoparticles with rumen fluid collected from cattle mixed with buffer solution, it was observed that the relative abundance of methanogenic archaea exhibited a dose-dependent decreasing trend with increasing nanoparticle concentrations. In a simulated dynamic rumen environment, compared with non-functionalised nanoparticles, the administration of functionalised nanoparticles resulted in a maximum reduction in methane production by 15% over 11 days, accompanied by a sustained increase in the concentrations of short-chain fatty acids such as butyrate. It should be noted, however, that these results were obtained from in vitro rumen fluid simulations, and the in vivo methane reduction efficacy of these nanoparticles in live ruminants remains to be validated through animal feeding trials. In addition, Chang et al. [110] prepared nanofibrous microcapsules encapsulating lactic acid bacteria using electrospinning technology. The microcapsules were first mixed with rumen fluid collected from Holstein dairy cows and dietary substrate, and incubated for 48 h. Subsequently, 0.1 N HCl solution containing 1 g/L pepsin was added to the fermentation bottles to simulate gastric fluid. After 1 h, the pH was neutralised with 1 N NaOH, and phosphate buffer containing 3 g/L pancreatin was added, followed by incubation for 2 h to simulate small intestinal digestion. The results demonstrated that direct addition of free lactic acid bacteria powder led to a substantial increase in bacterial abundance in the rumen, whereas the encapsulated lactic acid bacteria were not released or proliferated in the rumen. Notably, the abundance of lactic acid bacteria in the intestine increased threefold, confirming that the microcapsules could successfully protect the bacteria through the rumen and release them in the intestinal tract. This study represents a typical application case of targeted delivery in ruminants, as it successfully demonstrates the key concept of “rumen protection followed by intestinal release” using a sequential in vitro digestion system that mimics the ruminant digestive tract. Li et al. [111] prepared sodium alginate–carboxymethyl chitosan hydrogel beads crosslinked with citric acid, in combination with a water-in-oil-in-water (W/O/W) emulsion. The hydrogel beads were first placed in simulated gastric fluid for 2 h and then transferred to simulated intestinal fluid for 5 h. It was found that the beads maintained their structural integrity in the simulated gastric fluid, with only minimal probiotic release. Upon transfer to the intestinal fluid, the beads underwent disintegration, accompanied by sustained probiotic release. These results demonstrated that the hydrogel beads could effectively protect the encapsulated cargo and achieve sustained release in the intestinal environment. In vitro simulation experiments allow for the evaluation of the responsive characteristics of targeted delivery materials under various physiological environments. By simulating the rumen, abomasum, and intestinal conditions, these experiments can verify whether the delivery materials can provide protection, sustained release, and targeted delivery of the encapsulated compounds, thus offering valuable reference data for assessing their protective efficacy. However, static culture systems fail to realistically mimic the dynamic flow and absorption processes of the digestive tract, and the lack of standardized parameters across different livestock and poultry species limits limited comparability. In the future, species-specific dynamic in vitro models should be established to improve predictive accuracy.

### 4.3. In Vivo Fluorescence Imaging

In the in vivo evaluation of targeted delivery materials in animals, conventional sampling methods are cannot dynamically visualize the transport behaviour of carriers within the animal body. In contrast, in vivo fluorescence imaging technology enables the direct visualisation of the distribution and targeted release characteristics of the delivery carriers in vivo. Zhang et al. [112] labelled cationic glycosylated nanoparticles with the lipophilic fluorescent dye DiD. Mice were orally administered equal amounts of free DiD and DiD-labelled nanoparticles, respectively, and the fluorescence distribution of the nanoparticles was compared. It was found that at 1 h post-administration, the fluorescence signal of the nanoparticles was the strongest in the proximal small intestine, while it decreased markedly in the large intestine, indicating that the nanoparticles could target the small intestine for release. In addition, Cheng et al. [113] encapsulated fluorescently labelled *Escherichia coli* within enzyme-triggered multilayer microcapsules and monitored the fluorescence signal distribution in mice after oral gavage using an in vivo imaging system. It was found that microcapsules with two layers of protamine exhibited the strongest fluorescence signal in the colon at 12 h post-administration, whereas no colonic fluorescence accumulation was observed for unencapsulated probiotics, confirming that the microcapsules could successfully protect the probiotics through the gastric acidic environment and achieve targeted release in the colon. However, for active substances that require efficient action in the proximal segments of the intestinal tract, prolonging the residence time of carriers in the small intestine is equally important. Li et al. [114] labelled lysozyme nanoparticles and their oxidised starch microgel composites with Cy5 or Cy7 fluorescent dyes, and administered them to mice by oral gavage. Using an in vivo fluorescence imaging system, the distribution of the gels in the gastrointestinal tract was observed. It was found that fluorescence signals could still be detected in the small intestine at 8–12 h post-gavage. In contrast to the free lysozyme nanoparticles, the fluorescence signal of the gel composites began to weaken after 3 h. The gel shell enhanced the adhesive function of the nanoparticles, successfully resisted gastrointestinal digestion, and prolonged the retention time of the carriers in the small intestine. Through fluorescent labelling of carriers, the targeted delivery process in different intestinal segments can be clearly visualised. It should be emphasized, however, that all the above studies were conducted in mouse models via oral gavage, and the results obtained represent preclinical evaluations rather than direct evidence for livestock production. Although in vivo fluorescence imaging technology provides in vivo technical support for the design and optimization of targeted delivery materials, fluorescent labels are prone to metabolic clearance or quenching in vivo, leading to diminished long-term tracking signals. Moreover, fluorescent probes may interfere with the surface physicochemical properties of carriers or trigger non-specific immune recognition. Therefore, it is necessary to combine ex vivo quantitative methods such as histology and liquid chromatography-mass spectrometry (LC-MS) to evaluate the targeting performance of carriers more comprehensively and objectively. However, current in vivo evaluations of targeted delivery materials still suffer from inherent limitations. Differences among animal species in gastrointestinal physiology, enzymatic activity, and microbial composition suggest that data obtained from a single animal model may not reliably predict the actual performance in other livestock species. Moreover, most studies remain limited to short-term feeding trials, and systematic evaluations regarding the long-term safety, metabolic behavior, and impacts on gut microbial homeostasis of these delivery materials are still relatively insufficient. Considering the high costs, prolonged durations, and ethical constraints associated with large-animal trials, future research should integrate standardized in vitro digestion models, intestinal function assessment techniques, and validation strategies across multiple animal models to establish a more comprehensive multi-tiered evaluation system. Such an approach would enhance the predictive accuracy of targeted delivery system performance and facilitate its practical application in animal production.

In addition to tracking carrier distribution, ex vivo histopathological examination serves as an indispensable complement to in vivo fluorescence imaging for comprehensively evaluating the biosafety and efficacy of targeted delivery systems. Histological analysis enables direct assessment of epithelial integrity, mucosal architecture, goblet cell distribution, and immune cell infiltration (e.g., neutrophils, macrophages, and T lymphocytes) at the target sites, thereby providing crucial evidence for whether the released cargo exerts local therapeutic or nutritional effects without inducing tissue toxicity [115,116]. For instance, increased goblet cell density and intact villus–crypt structures in intestinal sections can corroborate the mucosal protective effects of encapsulated bioactive compounds, while reduced inflammatory cell aggregation validates their anti-inflammatory capacity [117,118]. Moreover, histology allows for the detection of potential adverse effects, such as epithelial erosion, edema, or granuloma formation, which may not be revealed by fluorescence signal alone. Integrating histopathological evaluation with particle size characterization, in vitro digestion models, and in vivo imaging offers a more robust and multidimensional framework for assessing carrier performance, from physicochemical properties to biological outcomes. This combined approach is particularly critical for long-term safety assessments and for translating targeted delivery technologies into practical animal production systems.

**Figure 3:**
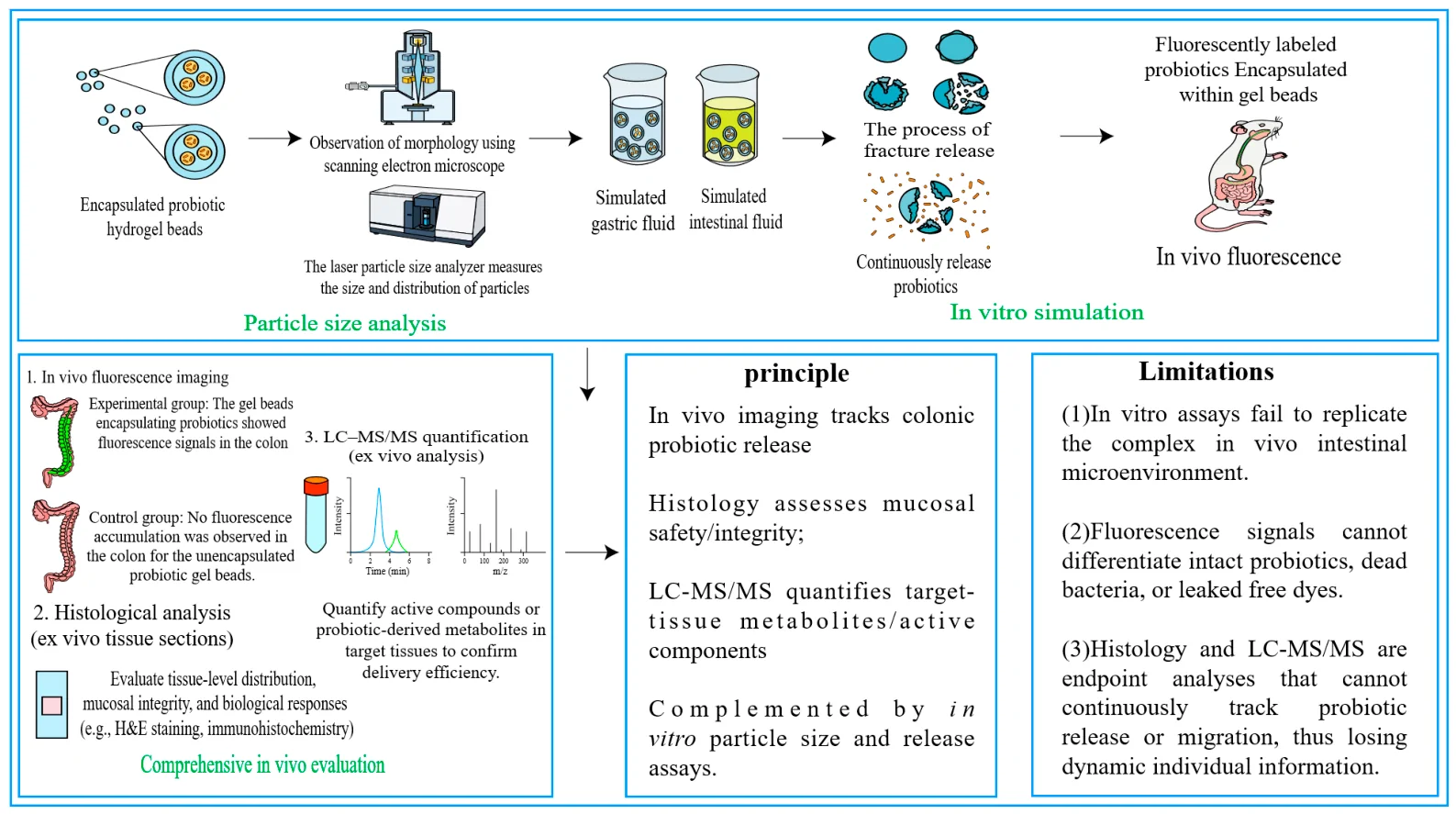
Characterization, in vitro release, and in vivo fluorescence evaluation of probiotic-encapsulated hydrogel beads.

Apart from evaluating successful targeting and release, unsuccessful delivery outcomes should also be considered during evaluation. Carriers may exhibit incomplete release, excessive retention, or unexpected biodistribution after administration. A high encapsulation efficiency does not necessarily indicate improved biological availability, because the encapsulated compounds may remain trapped within the carrier matrix or fail to reach the intended physiological site. Therefore, future evaluation systems should incorporate parameters such as carrier degradation rate, residual carrier accumulation, inflammatory responses, and functional activity of released compounds. Overall, the evaluation methods for targeted delivery materials each have their own advantages and limitations. Particle size analysis can assess the homogeneity and structural integrity of carriers but cannot reflect their functionality in complex biological environments. In vitro simulation enables evaluation of the protective and release effects of carriers on encapsulated compounds, yet it struggles to replicate dynamic in vivo processes. In vivo fluorescence imaging allows for direct visualization of targeted distribution and release, but it relies on fluorescent labelling, which may suffer from signal attenuation, environmental interference, and potential toxicity risks, in addition to high operational complexity and cost. The evaluation methods, their assessed parameters, principal advantages, and inherent limitations are summarized in Table 5. As shown in Figure 3, the characterization of particle size and morphology, in vitro release profiles, and in vivo fluorescence evaluation results of probiotic-loaded gel beads collectively demonstrate the complementary application of these methods. The three approaches complement each other, and their combined use facilitates a more comprehensive assessment of the performance of targeted delivery materials.

## 5. The Application of Targeted Delivery Technology in Animal Production

### 5.1. The Application of Targeted Delivery Technology in Poultry Production

Intestinal parasites represent a significant threat to poultry health. Conventional control methods are associated with issues such as drug residues and antimicrobial resistance. Targeted delivery technology can transport drugs to gut-associated lymphoid tissues (GALT), induce mucosal immune responses, and achieve specific elimination of parasites. Haseeb et al. [119] prepared chitosan nanoparticles loaded with recombinant Em14-3-3 protein and administered them via intramuscular injection into the thigh region of laying hens. The results showed that, compared with the control group, the immunised group exhibited significantly increased proportions of CD4^+^/CD3^+^ and CD8^+^/CD3^+^ T cells in the spleen, as well as elevated serum levels of IgY antibodies and IFN-γ cytokine concentrations. The nanoparticles likely delivered the antigen to lymphoid tissues capable of activating T cells, thereby eliciting a stronger cellular immune response. In addition to injection, oral targeted delivery also holds considerable potential for improving poultry health, as it can enhance the enrichment and transmucosal transport of encapsulated compounds through intestinal absorption. Wangngae et al. [120] synthesised glucose-modified lipid-based nanoparticles and administered them to chickens by oral gavage. A significantly enhanced Cy5 fluorescence signal was detected in the ileal tissue, showing a 27-fold increase compared with the control group. The enrichment of the nanoparticles in various segments of the small intestine was also markedly elevated, demonstrating that glucose-modified nanoparticles could achieve targeted enrichment and facilitate transmucosal transport in the chicken small intestine.

Targeted delivery technology has also been applied to plant extracts. Tannins are plant-derived bioactive compounds with antioxidant and antibacterial activities; however, they are susceptible to degradation by gastric acid and are difficult to reach the distal small intestine, leading to low bioavailability through conventional administration. Tian et al. [121] were the first to incorporate microencapsulated hydrolysed tannins into the diets of Zhongshan ducks. The results showed that supplementation with 400 and 800 mg/kg of microencapsulated hydrolysed tannins was associated with upregulation of *PPARγ*, *FAS*, and *LPL* genes in the breast muscle, suggesting that the microcapsules released tannins in the distal small intestine, where they activated the PPARγ signalling pathway. By activating the PPARγ signalling pathway, the microcapsules promoted fatty acid synthesis and uptake, thereby improving intramuscular fat deposition and meat quality, while also enhancing the antioxidant capacity and growth performance of Zhongshan ducks. Adaszyńska-Skwirzyńska et al. [122] prepared calcium alginate hydrogel capsules encapsulating lavender essential oil and administered them to chicks. Compared with the control group that did not receive the hydrogel, the abundance of *Christensenella* in the caecum of chicks was significantly increased, while the abundance of *Erysipelothrix* was significantly reduced, and body weight gain was also improved. These findings are consistent with the hypothesis that the hydrogel-encapsulated essential oil could deliver the active compounds to the caecum, increase beneficial bacterial abundance, reduce pathogenic bacterial abundance, and enhance growth performance in chicks.

In addition to plant extracts, microencapsulated organic acids have also been investigated as effective targeted delivery systems in broiler production. Nazir et al. [123] evaluated the effects of dietary supplementation with microencapsulated butyric acid (EBA) and yeast culture (YC), alone or in combination, on broiler performance and gut health. The results demonstrated that the combined EBA and YC treatment significantly improved body weight gain and feed conversion ratio compared with the control and antibiotic growth promoter groups. Moreover, this combination notably enhanced villus height and the villus height-to-crypt depth ratio in the duodenum, indicating improved intestinal absorptive capacity. In terms of intestinal microbiota, birds receiving EBA and YC exhibited a significantly reduced ileal abundance of *Escherichia coli* and *Salmonella*. Additionally, the EBA + YC group showed increased relative weights of immune organs (spleen and bursa of Fabricius) and elevated antibody titers against Newcastle disease virus, suggesting enhanced humoral immunity. These findings indicate that microencapsulated butyric acid, particularly when combined with yeast culture, can serve as a promising alternative to antibiotic growth promoters in poultry, acting through the synergistic improvement in intestinal morphology, microbiota modulation, and immune function. Beyond improving intestinal health and growth performance under normal conditions, targeted delivery systems have also demonstrated efficacy in mitigating environmental stress in poultry. Abudabos et al. [124] investigated the effects of nano-emulsified vegetable oil (NEVO) and betaine supplementation via drinking water on broiler chickens subjected to cyclic heat stress (35 °C for 8 h daily). The nano-emulsified vegetable oil, a nanoscale delivery system designed to improve lipid solubility and absorption, significantly improved the average daily gain and feed conversion ratio of heat-stressed broilers during the finisher period (days 21–35). Although NEVO showed an intermediate effect on breast meat pH and temperature, its positive impact on growth performance highlights the potential of nanoemulsion-based delivery strategies to enhance energy utilization under adverse environmental conditions. This study provides evidence that nanoencapsulation technologies can be effectively applied not only to bioactive compounds but also to fundamental dietary energy sources, thereby offering a practical nutritional strategy to alleviate the negative impacts of heat stress in poultry production. Targeted delivery technology in poultry farming has the potential to help address the threat of intestinal parasites, mitigate the issues of drug residues and antimicrobial resistance associated with conventional treatments, and offer new strategies for improving poultry health, meat quality, and growth performance. The key application cases of targeted delivery technology in poultry production, along with their carrier types, targeting sites, and main outcomes, are summarized in Table 6.

### 5.2. The Application of Targeted Delivery Technology in Pig Production

Weaned piglets often possess an immature immune system, and the highly acidic environment and digestive enzymes in the gastrointestinal tract cause the degradation of orally administered immunomodulators, preventing them to reach the intestinal mucosa and exert their effects. López-Cano et al. [125] encapsulated IL-1β cytokines in protein nanoparticles and administered them orally to piglets. The resulting increasing trend of TNF-α concentration in the blood suggested that the nanoparticles reached the intestinal mucosa, thereby activating the immune system and improving the immunity of weaned piglets. However, although encapsulating cytokines in nanoparticles can protect them from gastrointestinal degradation, the lack of active recognition capability for specific immune cells makes it difficult to ensure precise activation of intestinal mucosal immune responses. To improve targeted delivery efficiency, antigens or immunomodulators can be directed to macrophages and dendritic cells in the intestine. On this basis, Li et al. [126] constructed thiolated sodium alginate gel microspheres, in which mannose-modified cationic liposomes loaded with porcine epidemic diarrhoea virus antigen and retinoic acid were encapsulated. These microspheres were orally administered to piglets. Through the targeting effect of mannose, the encapsulated cargo was inferred to have been delivered to macrophages and dendritic cells in the small intestine, significantly increasing IgA levels and antigen-specific IgG antibody titres, thereby enhancing mucosal immune responses and affording stronger protective effects in piglets after oral administration.

Many natural plant bioactive compounds possess antioxidant, anti-inflammatory, and immunomodulatory activities, yet their oral bioavailability remains low. To improve their efficacy, Moniruzzaman et al. [72] administered prepared curcumin nanospheres to finishing pigs. The results showed that jejunal villus height and goblet cell count were significantly increased, suggesting that the curcumin nanospheres successfully reached the intestinal mucosa and released curcumin, thereby promoting intestinal epithelial cell development and differentiation. Additionally, TNF-α expression was downregulated, while IgA and claudin-3 expression were upregulated, pointing to suppression of inflammation and improvement in intestinal immunity. Organic acids, as potential alternatives to antibiotics, have the ability to inhibit intestinal pathogens and improve growth performance. However, unprotected organic acids are rapidly absorbed in the stomach and duodenum, making it difficult for them to reach the hindgut, thus diminishing their antibacterial efficacy. Microencapsulation technology can protect organic acids against gastric acid and achieve targeted delivery to the distal intestine. Muniyappan et al. [127] incorporated microencapsulated organic acids as feed additives into the basal diet of finishing pigs, which significantly reduced the *Escherichia coli* count in faeces. Compared with unencapsulated organic acids, the microencapsulated organic acids were inferred to have been delivered to the distal small intestine and even the large intestine, thereby acting in regions where *E. coli* proliferates extensively and effectively inhibiting its growth. Targeted delivery can successfully address the issues of degradation and rapid absorption of active ingredients in the gastrointestinal tract, and holds broad application value in immune modulation, intestinal development, and antimicrobial strategies in pigs. Future research should conduct in-depth investigations into the role of targeted delivery technology in maintaining the health of piglets, with particular attention to weaning-induced diarrhoea, the stability of intestinal microbiota, and feed intake, as these factors collectively determine the success of nutritional interventions during the critical post-weaning period. A comparison of the application cases of targeted delivery technology in pig farming is presented in Table 7.

### 5.3. The Application of Targeted Delivery Technology in Ruminant Production

The rumen is an extensive fermentation system in which a substantial number of bioactive substances are degraded by microorganisms, leading to reduced feed utilization. Although the rumen can digest crude fibre, it also degrades proteins and other bioactive compounds. To improve the utilisation efficiency of active substances, Wu et al. [128] prepared compound probiotic microcapsules encapsulating *Bacillus coagulans* SN-8 and *Saccharomyces boulardii* SN-6. After feeding to dairy cows, it was found that the abundances of Firmicutes, Bacteroidetes, and Actinobacteria in the intestinal microbiota were significantly increased, while those of Proteobacteria and Spirochaetes were significantly decreased. The increase in beneficial bacteria and the reduction in harmful bacteria suggest that the microcapsules likely delivered the probiotics to the distal intestinal tract of dairy cows for release. However, it should be noted that this conclusion was drawn indirectly from changes in faecal microbial composition, rather than from direct evidence such as fluorescent labelling of the microcapsules or quantification of released probiotic content in the distal intestine. Future studies would benefit from incorporating such direct detection methods to confirm the precise site and extent of probiotic release in the gastrointestinal tract of dairy cows. However, the effectiveness of encapsulation in protecting bioactive compounds from ruminal degradation is highly dependent on the physicochemical properties of the wall material. While some carriers have shown success in delivering probiotics to the intestine, other materials may fail to withstand the harsh ruminal environment. For instance, gelatin, a commonly used encapsulating agent, is hydrophilic and prone to swelling and mechanical disruption in the rumen, which could compromise its protective function. Pena et al. [129] prepared gelatin capsules encapsulating fish oil and supplemented them into the diets of lactating dairy cows (with some treatments administered directly via ruminal fistula) to evaluate their rumen-protective effect. The results showed that although the concentrations of certain unsaturated fatty acids (e.g., trans-C18:1 isomers and DHA) in ruminal fluid and milk fat increased to some extent following feeding of the encapsulated fish oil, the capsule protection did not substantially enhance the target fatty acid levels. Further in vitro experiments revealed that the capsules increased in weight by 40% and decreased in compressive strength by 84% after exposure to rumen fluid, indicating that the capsule shell ruptured due to water absorption and mechanical abrasion, providing only limited retardation of fish oil release in the rumen and failing to achieve effective rumen protection. Future efforts should focus on reducing direct contact between capsules and ruminal water, such as through surface coating modifications, to improve their ruminal stability.

Ino et al. [130] developed rumen-protected lysine microcapsules using beeswax and carnauba wax as wall materials and lysine as the encapsulant, employing a fusion–emulsification method, and supplemented with 0–3% natural tannin as a protective enhancer. The rumen-protective effect was evaluated through in vitro rumen fermentation tests and nylon bag degradation assays in rumen-fistulated sheep. The results demonstrated that the addition of tannin improved the microencapsulation yield and efficiency, with the carnauba wax + 3% tannin treatment achieving the highest microencapsulation efficiency. Differential scanning calorimetry analysis revealed that lysine exhibited higher thermal stability in the wax matrix than in its free form, confirming the protective effect of the wax wall materials. Further in vitro fermentation and nylon bag degradation tests demonstrated that the carnauba wax + 3% tannin group had the lowest ruminal degradation rates and the highest retention of dry matter and crude protein, while ruminal pH and temperature remained within normal physiological ranges (pH 6.0–7.2, temperature of 38.5–39.3 °C) across all treatments, indicating that the wax microcapsules did not adversely affect the rumen fermentation environment. The microcapsule system using carnauba wax as the wall material with 3% natural tannin addition effectively protected lysine from ruminal degradation and improved its rumen-bypass rate, as determined by the in vitro fermentation and nylon bag degradation assays, offering a feasible technical approach for the development of rumen-protected amino acid preparations for ruminants.

Targeted delivery technology can protect encapsulated compounds from degradation in the rumen, achieve precise targeting to specified sites in the intestine, and enhance the utilization efficiency of encapsulated substances, thereby providing an effective strategy for improving feed utilisation, treating diseases, and ensuring the safety of animal products. However, research on targeted delivery technology in ruminants still faces numerous challenges. It is necessary to design carrier materials that can stably exist in the intestinal tract and achieve precise release, tailored to the physicochemical properties of the active substances, while also assessing whether the carrier wall materials have any adverse effects on rumen microorganisms. In the future, it will be essential to develop responsive delivery systems based on the segmental characteristics of the ruminant digestive tract and to systematically evaluate their impacts on ruminal microbial communities, thereby ensuring the safety and precision of targeted delivery technology. Table 8 summarizes the representative application cases of targeted delivery technology in ruminant production.

### 5.4. Challenges and Opportunities for Practical Application: Regulation, Scale-Up, and Animal Welfare

Targeted delivery technologies not only improve nutrient utilization and productive performance but also offer new avenues for enhancing farm animal welfare by promoting health and reducing the need for manual interventions. In certain cases, the administration of active substances—such as probiotics, plant extracts, or therapeutic agents—still depends on repetitive manual procedures, including oral gavage or injection. Frequent handling and restraint can provoke stress responses, thereby compromising animal welfare [131]. In contrast, targeted delivery systems, owing to their controlled-release properties, targeted accumulation capacity, and high bioavailability, can substantially reduce the effective dose and administration frequency while preserving bioactivity. Consequently, they minimize unnecessary animal handling and alleviate management-related stress at its source.

At the level of health management, targeted delivery technologies offer unique advantages in disease prevention by restoring intestinal barrier function, modulating the local immune microenvironment, and enhancing stress resilience. Previous studies have demonstrated that encapsulated probiotic formulations, plant-derived bioactive polyphenol delivery systems, and bioactive peptide carriers can effectively improve the intestinal morphology of livestock and poultry, enhance antioxidant enzyme activities, and reduce the expression of inflammatory cytokines [94], thereby decreasing the risk of herd outbreaks and reducing reliance on antibiotics or therapeutic drugs. Notably, the advanced in vitro gastrointestinal simulation models described above in this review provide practical technical support for implementing the 3R principles (Replacement, Reduction, Refinement). These in vitro models enable efficient screening of delivery materials for stability, release kinetics, and permeability in simulated gastrointestinal fluids prior to in vivo experiments, thereby reducing the need for animal testing during material optimization and minimizing the number of treatment groups and sample sizes in subsequent formal animal studies [132]. Coupling precision delivery strategies with high-fidelity in vitro evaluation platforms not only helps reduce research and development costs but also represents a necessary direction for animal science to balance productivity with ethical practices in the future.

Beyond technical effectiveness and scalability, the regulatory environment constitutes another critical factor influencing the practical application of targeted delivery materials as feed additives. In major livestock production markets, such as the European Union, the United States, and China, novel feed additives are generally required to undergo systematic safety and efficacy evaluations to ensure their safety for target animals, consumers, and the environment. Some natural polymeric carrier materials, such as alginates, chitosan, and pectin, have been widely used in food and biomedical fields owing to their excellent biocompatibility and long history of food application [133]. However, nanoscale delivery systems possess unique physicochemical properties that necessitate further elucidation of their long-term in vivo behaviour, absorption and metabolic processes, as well as their potential impacts on gut microbiota and environmental safety [134,135]. Currently, dedicated regulatory guidelines specifically addressing complex targeted delivery carrier systems in animal feed remain relatively limited, which to some extent increases uncertainty for commercial application. Therefore, establishing standardized in vitro and in vivo safety evaluation systems, along with developing regulatory guidance principles tailored to the specific characteristics of feed applications, is essential for advancing targeted delivery technologies from experimental research towards sustainable livestock production.

The practical translation of targeted delivery systems into animal production necessitates a comprehensive safety assessment that extends beyond demonstrating efficacy in the target species. Such an evaluation should systematically address four principal dimensions: safety for the target animal; consumer safety with respect to potential residues; occupational exposure risks for workers involved in feed manufacture and handling; and environmental consequences following excretion and disposal.

For the target animal, particular scrutiny should be directed towards dose-dependent effects, gastrointestinal stability and biotransformation, as well as the absorption, distribution, metabolism, and excretion (ADME) profiles of both the active compound and its carrier. This consideration is especially critical for nanoscale delivery systems, whose biological behaviour is governed not solely by chemical composition but also by physicochemical attributes such as particle size, surface characteristics, aggregation state, dissolution rate, and degradation kinetics [136,137]. Consequently, nanospecific physicochemical characterization and exposure assessment may be required as adjuncts to conventional toxicological evaluations [136].

From a food safety perspective, the potential transfer of active compounds, their metabolites, degradation products, or intact nanoscale fractions from feed into edible tissues, milk, or eggs necessitates careful evaluation. Particular emphasis should be placed on residue depletion dynamics and, where pharmacologically active substances are involved, the scientific establishment of appropriate withdrawal periods. The assessment must also consider whether the delivery carrier remains intact following gastrointestinal transit or undergoes dissolution and degradation, as these processes critically influence systemic exposure and, consequently, the risk profile for consumers [137,138]. Importantly, the safety profile of targeted delivery materials should not be assumed to be uniform across different carrier classes. Food-grade, biodegradable polymers—such as polysaccharides and proteins—are anticipated to undergo digestion, degradation, or excretion after fulfilling their delivery function. In contrast, persistent inorganic or otherwise novel nanoscale materials may exhibit distinct patterns of gastrointestinal persistence, tissue interaction, environmental fate, and potential for bioaccumulation [136,139]. Therefore, risk assessment strategies must be tailored to the specific composition, physicochemical characteristics, degradation behaviour, dosage, and intended route of exposure for each delivery system.

Occupational safety represents an additional and frequently overlooked consideration, particularly during the manufacture, premixing, transportation, and on-farm application of feeds containing novel delivery materials. Dry powders and nanoscale materials, in particular, may generate inhalable or dermally exposed fractions during handling, with the magnitude of such exposure potentially changing substantially during scale-up from laboratory preparation to industrial feed production. Accordingly, user exposure characterization and the implementation of appropriate risk-management measures should be integral components of the safety evaluation for novel feed additives and their associated delivery systems [140].

Environmental safety must be considered across the entire life cycle of targeted delivery systems. Following administration, active compounds, metabolites, carrier degradation products, or persistent particulate fractions may be excreted in manure and subsequently partition into terrestrial or aquatic compartments. The environmental behaviour of engineered nanomaterials can vary considerably as a function of particle composition, size, surface modification, and prevailing environmental conditions, with reported effects on non-target organisms and potential for bioaccumulation warranting further investigation [139,141]. By contrast, readily biodegradable food-grade carriers are expected to exhibit different environmental persistence and transformation profiles; nevertheless, their environmental safety should still be verified under realistic exposure scenarios.

In conclusion, future regulatory frameworks should adopt a material-specific, exposure-driven paradigm that integrates target-animal safety, consumer exposure and residue depletion, occupational exposure, environmental fate and toxicity, alongside the detailed physicochemical and degradation characteristics of the delivery carrier. Such a holistic and scientifically robust framework would provide a more reliable basis for determining the safety and regulatory feasibility of targeted delivery technologies prior to their large-scale application in animal production.

## 6. Conclusions and Future Viewpoints

In contrast to previous reviews that typically focus on a single carrier category, this review provides a comparative framework integrating nanoparticles, microcapsules, and gels for animal production, offering a systematic comparison of their preparation methods, encapsulation performance, and applicability across species. However, the available evidence regarding real-world performance remains markedly uneven—conventional systems such as alginate-based microcapsules and lipid-coated rumen-protected nutrients have relatively more in vivo data in poultry, pigs, and ruminants, whereas most advanced nanocarriers are still supported by preclinical or in vitro studies, with limited validation under commercial feed processing (e.g., pelleting, extrusion, and long-term storage) and scarce data on long-term safety, tissue residues, and ecotoxicological consequences. Although targeted delivery systems show promising potential for improving intestinal health, immune function, pathogen inhibition, and growth/product quality, the majority of carrier materials are directly adopted from pharmaceutical or food science fields, with insufficient development of specialized systems tailored to the ruminant gastrointestinal tract—particularly the rumen-protective challenge, which remains a critical bottleneck that has been largely overlooked in previous reviews and demands dedicated material design and evaluation. Future research should prioritize livestock-specific, cost-effective, and process-compatible carriers, especially for ruminants, while optimising scalable preparation, developing standardized in vitro/in vivo evaluation platforms, performing comprehensive long-term safety and residue assessments, and clarifying carrier–microenvironment interactions. Extending these technologies towards low-carbon farming, functional animal products, and novel green feed additives could offer technical pathways for precision and sustainable livestock production, provided that the aforementioned knowledge gaps and safety uncertainties are adequately addressed.

## Figures and Tables

**Table 1:** Targeted delivery vectors based on nanoparticles: Comparison of preparation methods, applicable substances, advantages and disadvantages.

Type of Targeted Delivery Materials	Encapsulating Substances	Wall Material/Substrate	Preparation Method	Advantages	Limitations
Nano-particles	Lutein	Chitosan/Sodium Alginate	Ionic gelation method	The preparation conditions are mild, which can prevent the degradation of the active substances and improve the bioavailability.	The encapsulation ability for some water-soluble active substances is limited
	Butyric acid and propionic acid	Poly(lactic-co-glycolic acid) (PLGA)	Emulsifying solvent evaporation method	Realize the encapsulation and controlled release of short-chain fatty acids (SCFA)	The colloidal suspension has poor stability and is prone to sedimentation
	PLGA nanoparticles	PLGA/chitosan	spray drying process	Improve the stability and processing tolerance of the powder	High temperatures may affect thermally sensitive substances
	Curcumin	Polyvinylpyrrolidone (PVP)/Chitosan (CS)	Nanoprecipitation	Fast preparation, low cost, excellent storage stability	After preparation, it is necessary to perform dialysis to remove the organic solvents. The process is rather cumbersome

**Table 2:** Targeted delivery vectors based on microcapsules: Comparison of preparation methods, applicable substances, advantages and disadvantages.

Type of Targeted Delivery Materials	Encapsulating Substances	Wall Material/Substrate	Preparation Method	Advantages	Limitations
Microcapsule	Plant essential oil	Sodium alginate/chitosan	Sharp hole coagulation bath method	Easy to operate and with low equipment requirements	It is usually of a single-layer structure and has limited protective effect
	Probiotics	Calcium alginate-based composite material	Ion cross-linking method	Can be constructed with multi-layer embedding structures.	Sensitive to mechanical shearing and environmental ionic strength
	Walnut oil	Sodium alginate/chitosan	complex coacervation	Normal temperature conditions, suitable for sensitive substances	Low mechanical strength
	Probiotics (*Lactobacillus pentosus*)	Sodium alginate/chitosan	Electrospinning technology	Significantly increase the survival rate of probiotics in simulated gastrointestinal fluid/Combine with prebiotics to enhance the protective effect	It has limited tolerance to mechanical external forces and high-salt ion environments, and its long-term storage stability is prone to be affected

**Table 3:** Gel-based targeted delivery carriers: Preparation methods, applicable substances, and advantages and disadvantages comparison.

Type of Targeted Delivery Materials	Encapsulating Substances	Wall Material/Substrate	Preparation Method	Advantages	Limitations
Nanogel	Enrofloxacin	Chitosan/Sodium Alginate	Ionic gelation method	Size can be precisely controlled	Need coaxial needles and N_2_ equipment
Gel microspheres	Fullerene alcohol	Low methoxy gellan/gelatin nanotube composite system	Oil-in-water emulsion combined with Ca^2+^ cross-linking method	The process is relatively simplified	The oil phase needs to be removed, and the process involves many steps
Microgel	Capsaicine	Sodium alginate/chitosan	Complex coacervation	Using the physical crosslinking method to avoid the residue of chemical crosslinking agents	Poor water solubility, low bioavailability; insufficient release stability, with gastrointestinal irritation properties
Hydrogel	*Lactobacillus plantarum*	Gelatin/Polyvinyl Alcohol	Enzymatic cross-linking method	High embedding efficiency, high freeze-drying survival rate, and no cytotoxicity	The enzymatic cross-linking conditions need to be optimized, and it is still possible for some extreme pH or enzyme environments to cause degradation

**Table 4:** Comparison of targeted delivery vectors by type, structure, materials and release mechanism.

Type	Range of Sizes	Typic Structure	Main Material	Main Release Mechanism	Main Feature
Nanoparticles	1–100 nm	Solid particle structure, without obvious core–shell interface	Chitosan, alginate, PLGA, lipids, polysaccharides, etc.	PH response, enzyme degradation, diffusion release, surface receptor recognition	Small in size, with a large specific surface area, it has good mucosal penetration ability, but its stability and processing tolerance are relatively weak
Microcapsules	1–1000 μm (Usually 1–100 μm)	Core–shell structure, including the core material and the external coating layer	Alginates, chitosans, pectins, proteins, lipids	Shell rupture, pH response, enzyme degradation, diffusion release	Highly effective in protection and suitable for targeted delivery to the gastrointestinal tract
Nanogel	20–200 nm	A nano-scale three-dimensional cross-linked hydrophilic polymer network that can absorb water and expand	Chitosan, hyaluronic acid, polyethylene glycol, polysaccharide	Network swelling control, stimulus-responsive release	Combining nanoscale size and gel responsiveness, it is suitable for precise controlled release.
Hydrogel	*Lactobacillus plantarum*	Gelatin/Polyvinyl Alcohol	Enzymatic cross-linking method	High embedding efficiency, high freeze-drying survival rate, and no cytotoxicity	The enzymatic cross-linking conditions need to be optimized, and it is still possible for some extreme pH or enzyme environments to cause degradation

**Table 5:** Comprehensive evaluation methods for targeted delivery materials in animal production: assessed parameters, advantages, and inherent limitations.

Evaluation Methodology	Key Parameters Assessed	Principal Advantages in Animal Studies	Inherent Limitations and Challenges
Particle size, distribution, and morphology	Mean diameter & polydispersity index (PDI) Surface topography & internal structure (SEM, TEM) Batch-to-batch uniformity	Fundamental quality control metrics for carrier reproducibility Determine mucosal penetration, cellular uptake, and circulation fate Relatively low-cost and standardized protocols	Static characterization fails to predict dynamic in vivo behavior Poor correlation with biological functionality in complex GI environments Limited ability to assess carrier stability during feed processing (e.g., pelleting, extrusion)
In vitro simulation	Structural integrity in simulated gastric/rumen fluids Release kinetics in intestinal fluid (pH/enzyme-triggered) Protection efficacy for viable probiotics or bioactive compounds	Enables high-throughput screening prior to in vivo trials (supports 3R principles) Allows segment-specific assessment (rumen-bypass, small intestine, colon-targeting) Cost-effective for mechanistic elucidation	Static batch systems cannot replicate dynamic flow, peristalsis, or absorption Species-specific GI parameters (pH, transit time, enzyme profiles) are poorly standardized Overestimates or underestimates in vivo release due to absence of systemic metabolism and immune clearance
In vivo fluorescence imaging	Real-time biodistribution along GI tract Site-specific accumulation and retention time Semi-quantitative signal intensity (e.g., Cy5, DiD labelling)	Allows longitudinal, non-invasive tracking in the same individual Visually confirms targeted release and mucosal localization Crucial for validating carrier design strategies	Fluorescence quenching and metabolic clearing limit long-term observation Fluorescent probes may alter carrier surface properties or elicit non-specific immune recognition Currently restricted to small-animal models (mostly rodents); large-animal translational data lacking High equipment cost and technical expertise required
Ex vivo histopathological examination	Epithelial integrity & villus–crypt architecture Goblet cell density and mucosal thickness Inflammatory cell infiltration (neutrophils, macrophages, T cells) Tissue toxicity (erosions, edema, granulomas)	Directly evaluates local biological efficacy and biosafety at target sites Complements imaging by confirming functional outcomes (e.g., barrier repair, anti-inflammation) Provides definitive evidence for intestinal health improvement	Invasive and terminal, preventing longitudinal studies in production animals Sampling bias due to tissue heterogeneity Semi-quantitative and subject to observer interpretation High costs and ethical constraints associated with large-animal trials

**Table 6:** Comparison of application cases of targeted delivery technology in poultry production.

Animal Category	Target Compound/Active Substance	Dose/Concentration	Method of Administration	Target Site	Carrier Type	Release/Targeting Mechanism	Study Design/Evidence Type	Duration	The Main Impacts on Animal Production/Health	Limitations
Chicken	Recombinant Em14-3-3 protein	100 μg per chicken	Intramuscular injection	The entire lymphoid tissue or the spleen	Chitosan nanoparticles	Injecting into the targeted lymphoid tissues and activating the T-cell immune response	Controlled immunization trial in poultry (in vivo)	Primary immunisation at 2 weeks of age, booster one week later, challenge at day 28, necropsy 6 days post-challenge (~6 weeks of age)	Significantly increase the ratio of CD4^+^/CD8^+^ T cells, serum IgY antibodies and IFN-γ levels, and enhance cellular immunity	Intramuscular administration causes severe stress and is not suitable for large-scale production. Only immune indicators were evaluated, and no direct protective effect against coccidian infection was observed
Chicken	Glucose-modified lipid nanoparticles	2.5 μmol/L Cy5 (cell assay); animal dose not reported	Oral gavage	Small intestine (especially the ileum)	Glucose-modified lipid nanoparticles	The glucose group-mediated active targeting promotes the uptake and transmembrane transport of intestinal epithelial cells	Controlled gavage trial in poultry (fluorescence tracing)	Sampling at 3 and 6 h post-administration	The fluorescence signal in the ileum tissue increased by 27 times, and the enrichment in each small intestinal segment significantly increased	This is for fluorescence tracing studies only. No actual drugs or nutrients have been encapsulated. The long-term oral intake effects on intestinal health are unknown
Duck	Hydrolysable tannin	400 and 800 mg/kg feed	Dietary supplementation	Distal small intestine	Microcapsule	PH/intestinal environment response, released in the distal small intestine	Controlled feeding trial in poultry	56 days	Upregulate the expression of *PPARγ*, *FAS* and *LPL* genes in the pectoral muscles; promote fatty acid synthesis; improve intramuscular fat deposition and meat quality; and enhance antioxidant capacity	The specific preparation method of microcapsules and the release curve have not been described in detail. The mechanism research has only reached the level of the gene
Chick	Lavender essential oil	Not reported (0.4 mL/L water, provided as hydrogel capsules for voluntary intake)	Dietary supplementation	Cecum	Calcium alginate hydrogel capsules	The gel protection is delivered through the upper digestive tract and is released at the cecum	Controlled feeding trial in poultry	35 days (1–35 days of age)	Increase the abundance of beneficial bacteria *Christensenella* in the cecum, reduce the abundance of pathogenic bacteria *Erysipelothrix*, and increase weight gain	The composition of essential oils is complex, and the specific active ingredients are unknown; the effects on intestinal morphology and immune indicators have not been evaluated
Broiler	Microencapsulated butyric acid (EBA) + Yeast culture (YC)	EBA: 0.3 g/kg feed; YC: 1 g/kg feed	Dietary supplementation	Duodenum/Intestinal mucosa	Microcapsule (for butyric acid); Yeast culture powder	EBA provides a slow release in the intestine via fat encapsulation; YC modulates gut microbiota and immunity via metabolites	Controlled feeding trial in broilers (in vivo)	35 days	Significantly improved BWG and FCR; increased carcass yield and breast muscle weight; enhanced VH (1776.2 μm) and VH:CD (7.30); increased NDV antibody titers and immune organ (bursa, spleen) weights; reduced ileal counts of *E. coli* and *Salmonella*	Synergistic mechanism between EBA and YC needs further elucidation; the molecular basis for improved performance and immunity requires more in-depth investigation; only a 35-day trial was conducted
Broiler	Nano-emulsified vegetable oil (NEVO) + Betaine (BET)	NEVO: 5 mL/L drinking water; BET: 2 g/L drinking water	Water supplementation	Systemic/muscle tissue	Nanoemulsion (NEVO); water-soluble additive (BET)	NEVO improves bioavailability of lipophilic components via nanoscale droplets, enhancing energy utilization; BET acts as osmoprotectant and methyl donor, maintaining cellular water balance	Controlled feeding trial in broilers (2 × 3 factorial design, in vivo)	21–35 days of age (14 days)	NEVO significantly improved ADG, FCR and PEF; BET increased breast fillet yield (30.8 → 32.6%) and improved pH_15min_ and pH_24hr_; both supplements partially mitigated heat-stress-induced growth depression	NEVO showed less pronounced effect on meat quality than BET; molecular regulatory mechanisms not deeply explored; relatively short trial period (14 days), long-term effects unknown

**Table 7:** Comparison of application cases of targeted delivery technology in pig farming.

Animal Category	Target Compound/Active Substance	Dose/Concentration	Method of Administration	Target Site	Carrier Type	Release/Targeting Mechanism	Study Design/Evidence Type	Duration	The Main Impacts on Animal Production/Health	Limitations
Piglet	IL-1β cytokine	20 μg total protein/kg BW per day (containing ~3.2 μg IL-1β/kg BW)	Oral gavage	Intestinal mucosa	Protein nanoparticles	Nanoparticles encapsulate cytokines to protect them from degradation in the gastrointestinal tract	Controlled pilot trial in piglets (in vivo)	7 consecutive days	The concentration of TNF-α in the blood is on the rise, and the immune system is activated	Lack of active targeting makes it difficult to precisely activate specific immune cells. Only a trend is observed, with no significant effect.
Piglet	PEDV antigen + retinoic acid	Not reported (PEDV at 10^5^ TCID_50_ per dose in mice; 2 mL per piglet in pigs)	Oral gavage	Small intestinal macrophages and dendritic cells	Mannose cationic liposomes + Thiolated alginate gel microspheres	Glycero-sugar actively targets antigen-presenting cells, and gel microspheres protect through the upper digestive tract	Controlled immunization and challenge trial in piglets	28 days (immunisation on days 0 and 14, challenge on day 28)	Significantly increase the levels of intestinal sIgA and serum antigen-specific IgG antibodies, and enhance mucosal immune protection	The preparation process is complex (with multiple layers of encapsulation), the cost is high, and it is difficult to achieve large-scale production
Growing and fattening pigs	Curcumin	CN1: 1.0 mL/kg diet; CN2: 2.0 mL/kg diet (curcumin concentration in CN formulation not specified)	Dietary supplementation	Intestinal mucosa (jejunum)	Curcumin nanospheres	Nanoencapsulation enhances solubility and oral bioavailability of hydrophobic curcumin, facilitating intestinal absorption and tissue distribution	Controlled feeding trial in growing-finishing pigs	40 days	Increase the height of intestinal villi and the number of goblet cells; down-regulate TNF-α, up-regulate IgA and Claudin-3, and improve intestinal development and immunity	The long-term effects of such additions on the growth performance of pigs (such as daily weight gain and feed conversion ratio, FCR) have not been clearly reported
Growing and fattening pigs	Organic acid mixture	0.05% and 0.10% of diet (500 and 1000 mg/kg feed, respectively)	Feed addition	The distal small intestine and large intestine	Microcapsule	The microcapsules protect it from being absorbed by the stomach and duodenum, and target it to the large intestine segment for release	Controlled feeding trial in growing-finishing pigs	16 weeks (grower: weeks 1–8; early finisher: weeks 9–12; late finisher: weeks 13–16)	Significantly reduce the count of *Escherichia coli* in feces and effectively inhibit the proliferation of pathogenic bacteria in the hindgut	The impact on beneficial gut bacteria (such as lactobacilli) was not evaluated, and the improvement effect on growth performance was not mentioned

**Table 8:** Comparison of application cases of targeted delivery technology in ruminant animal production.

Animal Category	Target Compound/Active Substance	Dose/Concentration	Method of Administration	Target Site	Carrier Type	Release/Targeting Mechanism	Study Design/Evidence Type	Duration	The Main Impacts on Animal Production/Health	Limitations
Dairy cattle	*Bacillus coagulans* SN-8 + *Saccharomyces boulardii* SN-6 (compound probiotics)	Low: 1 g/cow/day (2 × 10^10^ CFU/g); Medium: 5 g/cow/day; High: 10 g/cow/day (2 × 10^11^ CFU/day)	Dietary supplementation	Distal intestine	Composite probiotic microcapsules	The microcapsules protect in the rumen and release at the intestinal site	Controlled feeding trial in dairy cows (in vivo)	28 days (milk and faecal samples collected on days 0, 7, 14, 21, 28)	Increase the abundance of beneficial bacterial phyla (firmicutes, bacteroidetes) in the intestine and reduce the abundance of harmful bacterial phyla (proteobacteria, spirilla)	Indirect evidence: It is inferred from changes in the fecal flora, without direct proof through methods such as fluorescence labeling, that it is released in the intestinal tract
Dairy cattle	Fish oil (rich in DHA)	Trial 1: 200 capsules/cow/day (28 g EPA + 13 g DHA/day); Trial 2: 180 capsules/cow/day (15.58 g EPA + 12.75 g DHA/day, actual consumption ~170 capsules)	Dietary supplementation	Rumen—Small Intestine (Rumen Protection)	Gelatin capsules	The physical barrier is designed to prevent the fish oil from being hydrogenated in the rumen.	Controlled feeding trial in dairy cows (in vivo) with repeated Latin square designs	Trial 1: 4 × 4 Latin square, 21-day periods; Trial 2: 3 × 3 Latin square, 21-day periods	The effect is limited: it only slightly increases certain unsaturated fatty acids in rumen fluid and milk fat, but overall it does not result in a significant difference.	Failure of protection: The capsule ruptured in the rumen due to water absorption and swelling, as well as mechanical wear, failing to achieve effective rumen protection
Sheep	lysine	3% tannin inclusion (based on wax mass) in carnauba wax + lysine formulation (CWLys_3_%); shell-to-core ratio 2:1	In vitro + in situ (nylon bag in rumen-fistulated sheep)	Rumen—Reticulum (Rumen Protection)	Waxy microcapsules (beeswax/palm wax + tannin)	The hydrophobic waxy barrier resists degradation by rumen microorganisms, and tannins are used to further enhance the protection	In vitro (DaisyII) + in situ (nylon bag in rumen-fistulated sheep) trial	In vitro: up to 2880 min (48 h); In situ: 22 days (17 days adaptation + 5 days collection), with incubation times up to 2880 min	The best treatment group (palm wax + 3% tannin) significantly reduced the rumen degradation rate and increased the rumen-undegradable fraction of dry matter and crude protein	Only in vitro and nylon bag methods were used for evaluation, lacking production performance data for live animals. The long-term effects of adding waxy substances on rumen microorganisms and animal health are unknown.

## Data Availability

No new data were created or analyzed in this study. Data sharing is not applicable to this article.

## Abbreviations

The following abbreviations are used in this manuscript:

TDDS	Targeted Drug Delivery Systems
SLNs	Solid Lipid Nanoparticles
NLCs	Nanostructured Lipid Carriers
PLGA	Poly(lactic-co-glycolic acid)
PVA	Polyvinyl Alcohol
PUFAs	Polyunsaturated Fatty Acids
PDI	Polydispersity Index
W/O/W	Water-in-Oil-in-Water
MTGase	Microbial Transglutaminase
SNX10	Sorting Nexin 10
ShRNA	Short Hairpin RNA
Mrna	Messenger RNA
IL-1β	Interleukin-1 beta
TNF-α	Tumor Necrosis Factor-alpha
SOD1	Superoxide Dismutase 1
SOD2	Superoxide Dismutase 2
PeiR	Archaeal virus lytic enzyme PeiR
PhaC	Polyhydroxyalkanoate synthase C39 peptidase
C39	peptidase C39 peptidase family
SEM	Scanning Electron Microscopy
TEM	Transmission Electron Microscopy
AFM	Atomic Force Microscopy
FTIR	Fourier Transform Infrared Spectroscopy
LC-MS	Liquid Chromatography–Mass Spectrometry
ANOVA	Analysis of Variance
HT29	HT29 Human Colorectal Adenocarcinoma Cell Line
DiD	1,1′-Dioctadecyl-3,3,3′,3′-Tetramethylindocarbocyanine
Cy5	Cyanine 5
Cy7	Cyanine 7
BW	Body weight
BWG	Body weight gain
VH:CD	Villus height-to-crypt depth ratio
GALT	Gut-associated lymphoid tissues
FCR	Feed conversion ratio
ADG	Average daily gain
SCFA	Short-chain fatty acids
DHA	Docosahexaenoic acid
EPA	Eicosapentaenoic acid
PEDV	Porcine epidemic diarrhea virus
PVP	Polyvinylpyrrolidone
CS	Chitosan
W/O	Water-in-oil
